# Measurement of amyloid formation by turbidity assay—seeing through the cloud

**DOI:** 10.1007/s12551-016-0233-7

**Published:** 2016-11-23

**Authors:** Ran Zhao, Masatomo So, Hendrik Maat, Nicholas J. Ray, Fumio Arisaka, Yuji Goto, John A. Carver, Damien Hall

**Affiliations:** 1 Research School of Chemistry, Australian National University, Acton ACT, 2601 Australia; 2 Institute for Protein Research, Osaka University, 3-1- Yamada-oka, Suita, Osaka, 565-0871 Japan; 3 College of Bio-resource Sciences, Nihon University, Chiyoda-ku, Tokyo, 102-8275 Japan

**Keywords:** Amyloid biophysics, Turbidimetric method, Amyloid aggregation kinetics, Data reduction, Nonlinear signal response

## Abstract

Detection of amyloid growth is commonly carried out by measurement of solution turbidity, a low-cost assay procedure based on the intrinsic light scattering properties of the protein aggregate. Here, we review the biophysical chemistry associated with the turbidimetric assay methodology, exploring the reviewed literature using a series of pedagogical kinetic simulations. In turn, these simulations are used to interrogate the literature concerned with in vitro drug screening and the assessment of amyloid aggregation mechanisms.

## Introduction

**Fig. 1:**
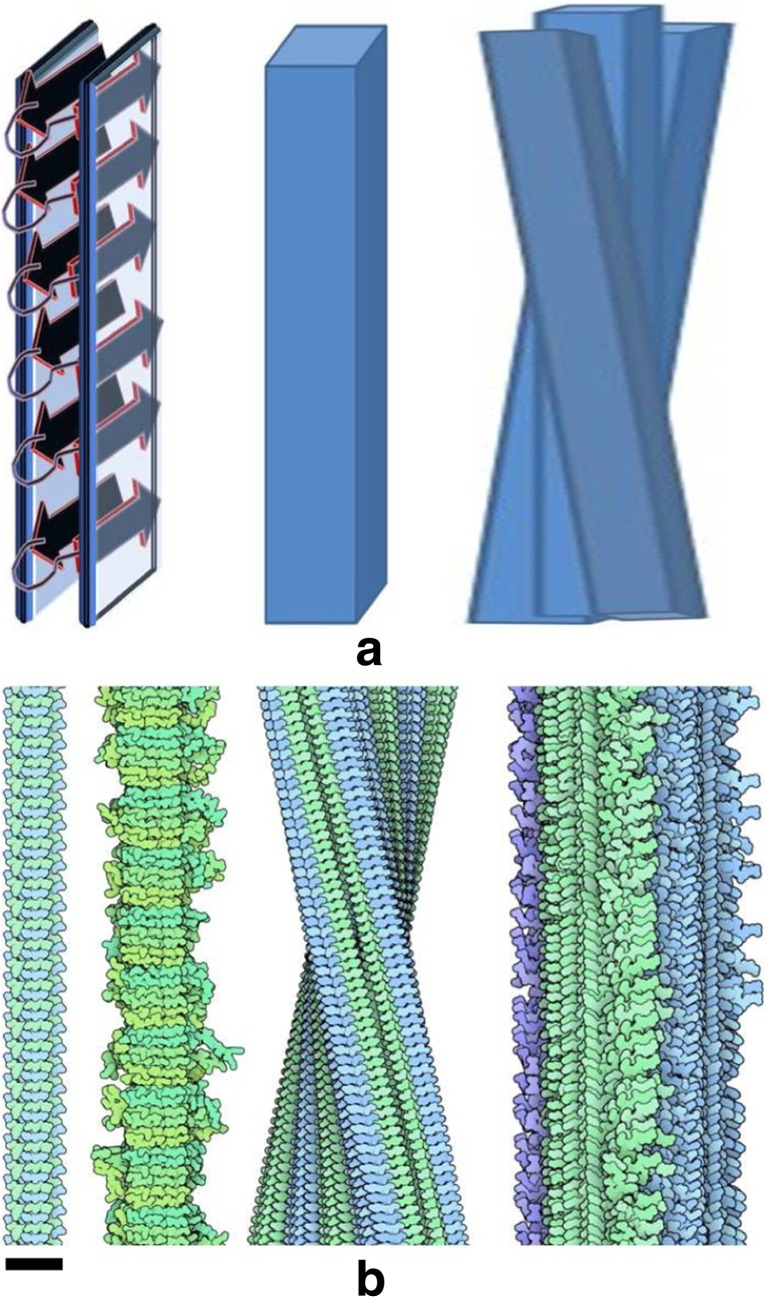
Amyloid structure.** a** Consensus structural features of the amyloid fibre.* Left* Intermolecular β-sheet stacks are formed between polypeptides along the direction of the fibre. One or more sections of a polypeptide may contribute to the longitudinal β-sheet formation. Hydrophobic-driven lateral packing may occur between the orthogonal faces of the β-sheet elements within the amyloid fibre.* Centre* The simplest possible fibre arrangement is termed a protofibril which can be characterized by a length, width, persistence length and helical pitch.* Right* Hydrophobic packing forces can cause multiple protofibrils to align to yield higher-order quaternary arrangements of amyloid fibres termed ‘mature fibres’ (figures adapted, with permission, from Hall and Edskes 2012).** b** Artistic renderings of the structures of four different amyloids solved by a combination of solid-state nuclear magnetic resonance and various types of electron microscopy.* From left to right* amyloid fibres derived from the human prion protein (Apostol et al. 2010), yeast prion amyloid fibres formed from the full-length yeast protein HET-s (Van Melckebeke et al. 2010), amyloid formed from a peptide segment of transthyretin (Fitzpatrick et al. 2013) and a mature amyloid fibre, composed of multiple protofibrils, derived from the brain of an Alzheimer’s Disease patient (Paravastu et al. 2008).* Bar in lower left hand corner* 5 nm [figures adapted from painted illustrations by D.G. Goodsell (Goodsell et al. 2015)]

The word ‘amyloid’ was first coined over 160 years ago to describe white densities of protein aggregate in autopsied livers, in the mistaken belief that they represented deposits of starch^1^ (Virchow 1854). In modern day scientific practice, the meaning of the term amyloid has extended beyond its original histopathological association with disease, to describe a class of nanofiber able to be formed by most proteins upon their adoption of an unfolded structure and subsequent polymerization via intermolecular β-sheet formation (Toyama and Weissman 2011; Tycko 2011) (Fig. 1). From this current perspective, amyloid is interpreted as a particular tertiary fold, whose structural maintenance is conditional upon its stabilization as part of a higher-order quaternary assembly.

Regardless of the motivation for its study, the most basic practical requirement for experimenting with amyloid is an assay procedure for monitoring its formation. Although there are numerous techniques that are capable of achieving this goal (Li et al. 2009; Nilsson 2004), by far the three most common in vitro assay formats are those based on turbidity (Dolado et al. 2005; Sant’Anna et al. 2016), induced fluorescence associated with Thioflavin T dye binding (Dalpadado et al. 2016; Levine 1993; Naiki et al. 1997) and induced absorbance spectral shift exhibited upon Congo Red dye binding (Klunk et al. 1989). In this review, we examine the literature concerned with the underlying theory and experimental interpretation of the turbidity assay (Andreu and Timasheff 1986; Moody et al. 1996). As such, our review differs from many others on the topic of amyloid biophysics (Hall and Edskes 2012; Kashchiev 2015; Ma and Nussinov 2006; Mezzenga and Fischer 2013; Sasahara and Goto 2013; So et al. 2016; Tycko and Wickner 2013) by its restriction to matters directly related to achieving an understanding of the turbidimetric method. Towards this goal our examination will pay particular attention to recent articles concerned with ultra-microscope image analysis (Hall 2012; Usov and Mezzenga 2015), light scattering and turbidity development by protein aggregates (Garcia-Lopez and Garcia-Rubio 2008; Garcia-Lopez et al. 2006; Hall et al. 2016a) and simulation of the kinetics of amyloid (Ghosh et al. 2010; Hall et al. 2016b; Kaschiev 2015) and other aggregate types (Adachi et al. 2015; Hall et al. 2015). Although placing the focus of the review on a single type of assay procedure may seem like a retreat from the bigger questions, such as the relation between amyloid and disease (Hall and Edskes 2012; Walker and Jucker 2015), we contend that a thorough understanding of principles associated with the turbidimetric monitoring of amyloid growth will sharpen our collective ability to make informed judgements about the biological implications of results gained from in vitro protein aggregation assays.

In the following sections we outline (1) consensus physical models of amyloid aggregates to better understand how they might interact with visible wavelength light, (2) the general physics of the interaction of light with matter, concentrating on the description of utilitarian mathematical transforms able to estimate the value of the turbidity on the basis of attainable experimental quantities and (3) consensus kinetic models of aggregate development capable of predicting broad features of the time course of aggregation for various limiting-case regimes of amyloid growth. As a means for summarizing relevant literature into compact review form, the geometric and turbidimetric transforms discussed in (1) and (2) are applied to the output of the consensus kinetic models presented in (3). These transformed data sets are then used as aids for the interpretation of literature related to amyloid aggregation kinetics.

## (i) Consensus physical models of protein aggregates

Solid-state nuclear magnetic resonance experiments and various electron microscopy techniques have been used, in combination, to determine atomic-level structural models for several amyloids (Tycko and Wickner 2013). Figure 1a is a schematic highlighting three consensus features displayed by nearly all amyloid structures observed to date (Tycko and Wickner 2013; Tycko 2014), namely:

1. intermolecular β-sheet formation directed parallel to the long axis of the fibre
2. hydrophobic stacking of β-sheet segments perpendicular to the long axis of the fibre (if more than one β-sheet motif is present per polypeptide)
3. lateral association of protofibrils to form multi-fibre assemblies

Figure 1b shows an artistic rendering by Goodsell (Goodsell et al. 2015) of four different amyloid structures formed from four different proteins, with all structures determined using hybrid-combination approaches (Apostol et al. 2010; Fitzpatrick et al. 2013; Lu et al. 2013; Van Melckebeke et al. 2010). Regular arrangements of stacked β-sheets can be seen in all rendered images. Packing restraints associated with these bonding patterns induce a differential diffraction of incident X-rays from fibres aligned perpendicularly to the incident radiation, with this image providing the basis of the ‘cross-β’ structural nomenclature^2^ often used to describe internal amyloid bonding patterns (Liu et al. 2016; Makin and Serpell 2005).

Although atomic models provide maximum structural information, they are often not representative of the population of amyloid fibres in typical in vitro, or in vivo, experiments, for which fibre heterogeneity tends to be the norm, rather than the exception (Guo and Akhremitchev 2006; Liu et al. 2016; Meinhardt et al. 2009). With regard to fibre structure, two general classes of variation exist. The first type of variation is related to heterogeneity in polymer length/width^3^ (Hall and Minton 2002, 2004; Hall et al. 2016b; Szavits-Nossan et al. 2014). Such variation in fibre length and width is a natural feature of any polymerization reaction capable of longitudinal growth and lateral association^4^ (Hall 2012; Ridgley and Barone 2013; Umemoto et al. 2014). The second type of variation is generated by intrinsic structural differences in the core amyloid/aggregate stemming from competing nucleation pathways available to a single polypeptide sequence (King and Diaz-Avalos 2004; Paravastu et al. 2008; Petkova et al. 2005; Tanaka et al. 2004; Toyama and Weissman 2011; Tycko and Wickner 2013). The availability of multiple aggregation pathways is thought to represent the basis of amyloid polymorphism (also known as strain formation) (King and Diaz-Avalos 2004; Tanaka et al. 2004; Tycko and Wickner 2013). Regardless of the cause of the variation, the end result is a heterogeneous mixture of fibres and other aggregate products (Hall 2012; Umemoto et al. 2014). Indeed, in order to be used in high-resolution structural studies, such heterogeneous fibre distributions must first be carefully treated by either selective degradation, purification or re-cultivation (via selection and re-seeding) in a manner analogous to crystal farming (Qiang et al. 2011; Scherpelz et al. 2016).

**Fig. 2:**
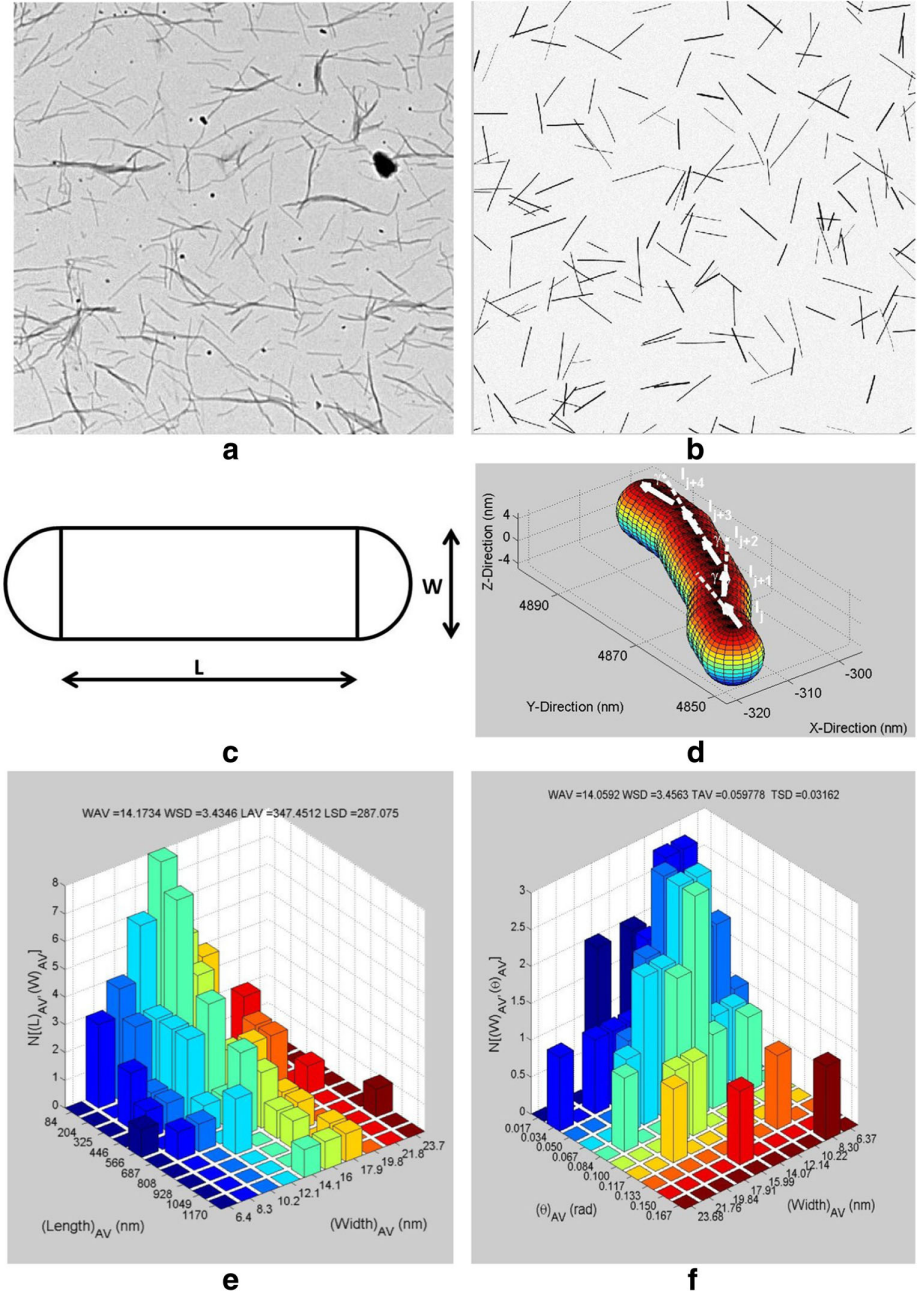
Ultramicroscopy-based analysis of protein aggregates can provide the necessary mesoscopic-level structural information for estimating turbidity via the methods outlined in the text of this review. **a** Typical experimental transmission electron microscopy (TEM) image of amyloid fibres (made from pig insulin at pH 3.0 and 60 °C, recorded at 6000× magnification (adapted, with permission, from Fig. 9 of Hall 2012). **b** Example of a pseudo-TEM image generated using the Amyloid Distribution Measurement (ADM) software useful for calibrating and testing image analysis routines and designing better ultramicroscope experiments (adapted, with permission, from Fig. 2 of Hall 2012). **c** Mesoscopic representation of fibre by a sphero-cylinder of variable internal length (*L*) and width (*W*) (adapted, with permission, from Fig. 8 of Hall 2012). **d** Average angle of deviation (θ_av_) for an individual fibre as determined by Hall et al. (2016a) using successive calculation of the dot product between projection vectors that trace along the backbone of the amyloid fibre (adapted, with permission, from Fig. 3a of Hall 2012). **e** Analysis of simulated TEM data yielding two-dimensional histograms of length and width (adapted, with permission, from Fig. 12 of Hall 2012). **f** Analysis of simulated TEM data yielding two-dimensional histogram of width and average deviation (adapted, with permission, from Fig. 12 of Hall 2012)

Atomic-level differences in amyloid structure are often invisible, or muted, when coarser assay methods are used (Li et al. 2009; Nilsson 2004). This is the case for amyloid scattering/turbidity experiments conducted using visible wavelength light (∼400–700 nm) for which the large wavelength—relative to the aggregate size—makes anything more than a mesoscopic^5^ description of amyloid structure superfluous. The most common and direct means for such estimations of mesoscopic structures involve the use of ultramicroscopy techniques, atomic force microscopy (Adamcik et al. 2010; Harper et al. 1997), transmission electron microscopy (TEM) (Goldsbury et al. 2011; (Hall 2012) or total internal reflection fluorescence microscopy (Ban et al. 2003, Ban and Goto, 2006)^6^. A small number of researchers (Hall 2012; Usov and Mezzenga 2015) have quantitatively reduced amyloid ultramicroscope images into equivalent hard particle models based on a description of the aggregates as spheres (for small amyloid/oligomers and large amorphous aggregates) or cylinders (for amyloid fibers) with the asymmetric bodies assigned a characteristic rigidity value (Adamcik and Mezzenga 2011; Hall 2012) or a defined chiral twist (Usov and Mezzenga 2015). In the study carried out by Hall (2012), semi-automated analysis software was developed and applied to TEM images of amyloid formed from bovine insulin under high temperature and low pH conditions (Fig. 2). In that work, two algorithms were used to reduce the fibre images to a table of characteristic values. The first algorithm (Eq. 1a, b, c) enabled deconvolution of the measured perimeter, P, and area, A, of an individual fibre (calculated from pixel analysis of the TEM image bitmap) in terms of an equivalent sphero-cylinder, with the result that each fibre was reduced to an internal length L and a fibre width W (Fig. 2c).

1a $$ P=2\left(L+\frac{\pi W}{2}\right) $$

1b $$ A=L\times W+\frac{\pi {W}^2}{4} $$

1c $$ \left(\frac{-\pi }{4}\right){W}^2+\left(\frac{P}{2}\right)W-A=0 $$

The second algorithm (Eq. 2; Hall 2012)– enabled the estimation of a quantity, θ_AV_, reflective of the linear persistence of amyloid fibres (Adamcik and Mezzenga 2011; Cantor and Schimmel 1980), defined as the average absolute deviation of the piecewise projection vector I, measured over a characteristic distance, *l*, selected as the fibre width^7^ (Fig. 2d).

2 $$ {\theta}_{AV}=\frac{1}{\left(N-1\right)}{\displaystyle \sum_{j=2}^N\left|{ \cos}^{-1}\left(\frac{{\mathbf{I}}_j.{\mathbf{I}}_{j-1}}{l_j.{l}_{j-1}}\right)\right|} $$

As shown in Fig. 2e, f, the analytical software developed by Hall (2012) is able to decompose fibre images into two-dimensional histograms of fibre length versus width or width versus average deviation. This approach was later used to analyze TEM images of size exclusion chromatography-purified amyloid fibres, thereby facilitating development of a quantitative theory of fibre elution by that technique (Hall and Huang 2012).

**Fig. 3:**
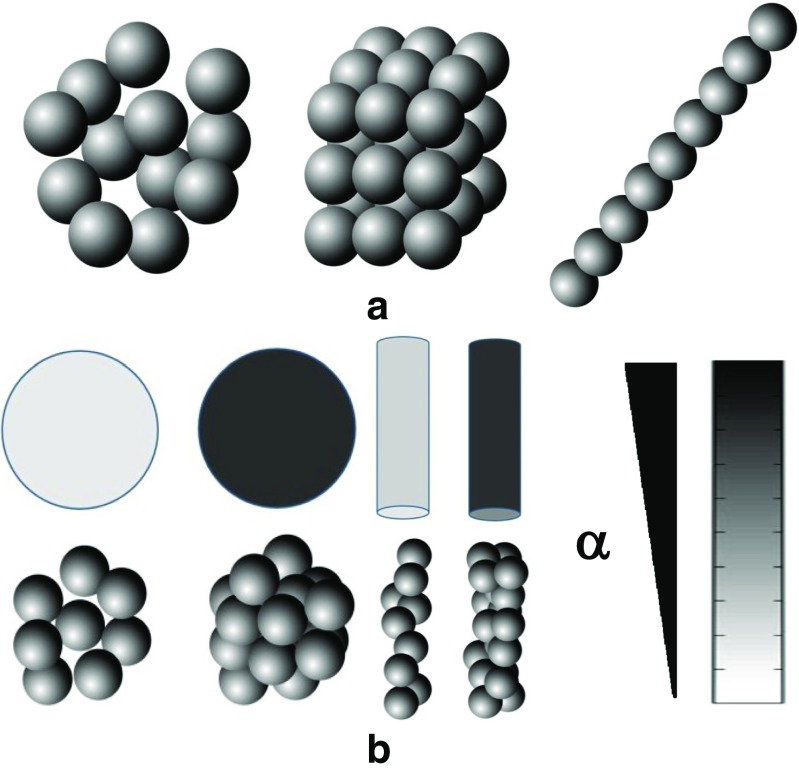
Coarse structural models of aggregates. **a** Schematic describing coarse-grained conceptualization of bonding arrangements seen in various types of protein aggregate corresponding to amorphous (*left*), crystalline (*middle*) and fibrous (*right*) structures. **b** Schematic describing mesoscopic structural groupings of aggregates as either rod-like or spherical with assignment of a volume packing fraction, defined by the parameter α, such that a* darker colour* represents a greater fractional occupancy of the aggregate trace volume by protein, i.e. a greater internal density (schematic is adapted, with permission, from Fig. 1 of Hall et al. 2016a, b)

An extension to this geometrical description, useful for modelling aggregates in solution (Hall et al. 2016a), involves representing aggregate geometry in terms of similarly limited shape possibilities, along with an extra variable relating to the internal volume packing fraction. Using this approach, amorphous, crystalline and fibrous protein aggregates can all be represented (Fig. 3). In this model, a protein aggregate composed of i monomers is defined by three properties, the molecular weight, M_i_, the shape, S_i_ and the volume trace (V_i_)_TRACE_ (Eq. 3a, b, c). With regards to the shape, S_i_, protein aggregates are treated as either arbitrarily diffuse rods, defined by a trace length L_i_ and a trace radius R_i_, or arbitrarily diffuse spheres, characterized solely by R_i_.

3a $$ {M}_i=i{M}_1 $$

3b $$ {\left({V}_i\right)}_{TRACE}=\frac{i{M}_1\upsilon }{\alpha_i{N}_A} $$

3c $$ {S}_i=\left\{\begin{array}{c}\hfill \mathrm{rod};\kern0.5em {L}_i={\left({V}_i\right)}_{TRACE}/\left(\pi {R_i}^2\right)\hfill \\ {}\hfill \mathrm{sphere};\kern0.5em {R}_i=\sqrt[3]{3\kern0.5em {\left({V}_i\right)}_{TRACE}/\left(4\pi \right)\ }\hfill \end{array}\right. $$

In Eq. 3, M_1_ describes the monomer molecular weight, N_A_ is Avogadro’s number, α_i_ is the fraction of the trace volume occupied by protein (Fig. 3b) and υ refers to protein partial specific volume^8^ (Lee et al. 2009). As noted (Hall et al. 2016a), defining α_I_ in the manner outlined by Eq. Eq. 3a, b, c allows it to be used to parameterize the transition between compact and diffuse aggregate states (α_DIFFUSE_ < α_COMPACT_ ≤ 1) such that a higher value of α would be more appropriate for crystal-like aggregates whereas a lower value would describe a less dense amorphous^9^ aggregate (Bennett 1972; Zurdo et al. 2001).

In principle, the geometrical information contained within an ultramicroscope image can be used to model the distribution of fibres within the solution from which it was generated. Although some research groups have made great strides forward (Arosio et al. 2012; Hall and Huang 2012; Lomakin et al. 1996; Rogers et al. 2005), this process is as yet a not fully realized proposition^10^. Here we take the liberty of pointing out how fibre shape parameters, derived from analysis of the ultramicroscope images, can be used to define a fibre trace volume. In conjunction with an assumed fractional volume packing, α_i_, the fibre molecular weight and degree of polymerization can be estimated from Eq. 4a, b, c.

4a $$ {\left({V}_i\right)}_{TRACE}=\left\{\begin{array}{c}\hfill \frac{4}{3}\pi {R_i}^3\kern1em \hbox{-}\ \mathrm{f}\mathrm{o}\mathrm{r}\ \mathrm{a}\ \mathrm{sphere}\hfill \\ {}\hfill {L}_i\pi {R_i}^2\kern1em \hbox{-}\ \mathrm{f}\mathrm{o}\mathrm{r}\ \mathrm{a}\ \mathrm{cylinder}\kern0.75em \hfill \end{array}\right. $$

4b $$ {M}_i={\left({V}_i\right)}_{TRACE}{\alpha}_i{N}_A/\upsilon $$

4c $$ i={M}_i/{M}_1 $$

To probe macroscopic-level phenomenon, one must be able to infer the concentration distribution of aggregate in solution based on knowledge of the number of adsorbed fibres, N, possessing properties within the discrete limits set by the element of a histogram^11^. For some techniques, such as light scattering (Lomakin et al. 1996; Nichols et al. 2002) or analytical ultracentrifugation (Binger et al. 2008), in which the total signal intensity is defined by the solution distribution, deconvolution can be attempted directly. However, for distributions inferred from ultramicroscope images, either an internal standard (Kirschner et al. 1975) or an independent measure of the total mass concentration of aggregate in solution, c_aggregate_, is required to determine absolute number densities (with c_aggregate_ defined as the total concentration of protein in an oligomeric form having a polymer degree ≥ 2). As shown previously (Borgia et al. 2013; O’Nuallain et al. 2006), a value of c_aggregate_ can be determined by pelleting or filtration assay, with concomitant spectrophotometric analysis of the supernatant. With this information available, the ultramicroscope image data can be converted into a solution distribution on the assumption that the derived distribution is a true representation of the solution state (Eq.5a, b).

5a $$ C\left(i\to i+\varDelta i\right)\kern0.75em =\kern0.75em \frac{N\left(i\to i+\varDelta i\right)}{{\displaystyle \sum_{j=2}^ZN\left(j+\varDelta j\right)}.\left(j+\frac{\varDelta j}{2}\right)}.{\mathrm{c}}_{\mathrm{aggregate}} $$

5b $$ C\left(i+\frac{\varDelta i}{2}\right)\kern0.75em =\frac{C\left(i\to i+\varDelta i\right)}{\varDelta i} $$

Equation 5a, b provides the means for inferring the solution aggregate distribution from ultramicroscope-derived histograms. Joining a continuous line between discrete concentration estimates (such as that provided by Eq. 5b) allows, in principle, for realization of the form of the fibre distribution in solution. In the next section, we examine the literature concerned with the estimation of the light scattering properties from such a protein aggregate distribution, summarizing germane concepts into a set of equations capable of directly transforming aggregate distributions into turbidity (at a given wavelength and path length).

## (ii) Turbidity of aggregates in the visible region

**Fig. 4:**
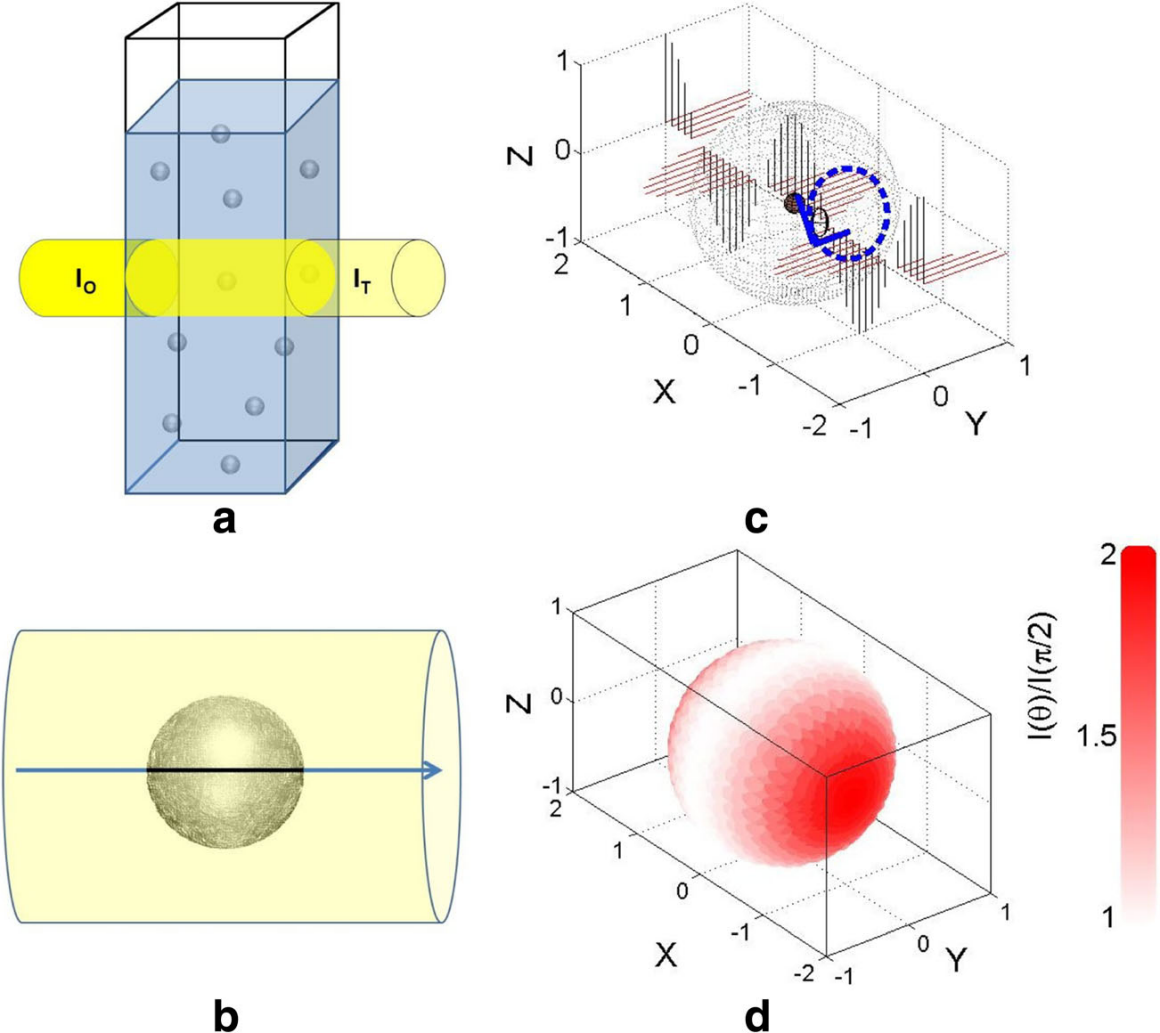
Principles of light scattering. **a** Schematic describing the transmission-based measurement of excess solution turbidity of protein aggregates in which the transmitted light intensity (*I*_*T*_) is measured in relation to the incident light intensity (*I*_*0*_) using a standard spectrophotometer (or plate reader). **b** Ray diagram of the encounter between light and the scattering particle in solution. **c** Simplified schematic of a general goniometric scattering experiment for non-polarized light (although the light wave shown has only one polarization!). Scattering intensity for Rayleigh-type scattering is equivalent when recorded at any point on a sphere (centre located at the scattering particle) defined by the radius (r) and the angle θ, whereby θ is defined as the sub-apex of the spherical solid angle measured from the forward scattering direction (adapted, with permission, from Fig. 2a of Hall et al. 2016a, b). **d** Colour plot indicating the scattering intensity (normalized relative to the scattering recorded at right angles to the incident beam) as a function of the recording angle θ, with the system conforming to limiting Rayleigh scattering conditions described in **c** (adapted, with permission, from Fig. 2b of Hall et al. 2016a, b)

Turbidity describes the attenuation of the incident beam by light scattering (Bohren and Huffman 2008; Elimelech et al. 2013) and thus can be evaluated either directly, via measurement of the loss of intensity by transmission measurement (Fig. 4a, b), or indirectly, by integration of the angle-dependent scattering at a fixed distance (goniometric static light scattering) (Doty and Steiner 1950; Wyatt 2014) (Fig. 4c, d). The relatively straightforward nature of the transmission measurement, requiring only a spectrophotometer or plate reader, has encouraged adoption of the turbidimetric method in the absence of more specialist light scattering equipment (Andreu and Timasheff 1986; Gaskin et al. 1974; Wyatt 2014). Coupled with ease of performance, the generality of light scattering (due to the lack of a requirement for an extrinsic label) has made transmission-based turbidimetric assays the default ‘basic’ standard for recording protein aggregation kinetics. Historically speaking, turbidity has been used to monitor the growth of a range of protein aggregation reactions, including helical fibre formation by sickle cell haemoglobin (Ferrone et al. 1985; Moody et al. 1996), cytoskeletal fibre formation (Voter and Erickson 1984; Wegner and Engel 1975; Gaskin et al. 1974), virus capsid formation (Tachibana et al. 1977), non-specific amorphous polymerization (Stranks et al. 2009; Ingham et al. 2011) and of course amyloid formation (Anzai et al. 2016; Dolado et al. 2005; Hatters et al. 2001; Necula et al. 2007; Sant’Anna et al. 2016). Although the turbidimetric procedure is a relatively non-demanding experiment to carry out, as with all scattering methods, the downside is that gaining an understanding of the underlying physics generally requires a familiarity with Maxwell’s equations not often in simpatico with the background of those performing the work. It is partly towards this divergence that the next section is directed.

The truest understanding of light scattering phenomena requires a quantum-level description of both the system and the light source (Chu 1974; Loudon 2000). However from the time of Rayleigh (Rayleigh 1899), theories based on classical electromagnetism (Bohren and Huffman 2008; Doty and Steiner 1950; Oster 1955; Penzkofer et al. 2007), coupled with regular shape approximations of the scattering bodies and continuum approximations of the solvent, have proven effective for extracting shape and molecular weight information from measurement of the angle-dependent intensity of the scattered light (Geiduschek and Holtzer 1958; Wyatt 2014). In the classical approach, light is considered to be a coincident, yet perpendicular pair of travelling electric and magnetic transverse field vectors, oscillating at a frequency f, over a wavelength λ (Bohren and Huffman 2008). The charge distribution associated with any element of matter in the path of the light beam is perturbed by these fields and caused to, itself, oscillate. For the case of elastic scattering, the oscillating (and thus accelerating) charge will produce another light wave of identical wavelength^12^ (Bohren and Huffman 2008; Kerker 2013). In the late 19th century, Strutt (Rayleigh 1899) deduced the quantitative relationship between the characteristics of an illuminated particle and the scattering intensity measured at a set distance and direction (Eq. 6a; Fig. 4). That formulation was derived on the basis of a set of simplifying criteria specifying limiting dilution and small size for the scattering object relative to the wavelength of light (Fig 4a). Under these Rayleigh limiting conditions the total amount of light scattered away from the forward direction, I_S_, can be calculated by spherical integration of the angle-specific scattering intensity, i(r,θ), whereby θ represents the forward scattering apical sub-angle of the solid angle and r describes the radial distance from the centre (Kerker 2013; Oster 1955) (Eq. 6b– e).

**Fig. 5:**
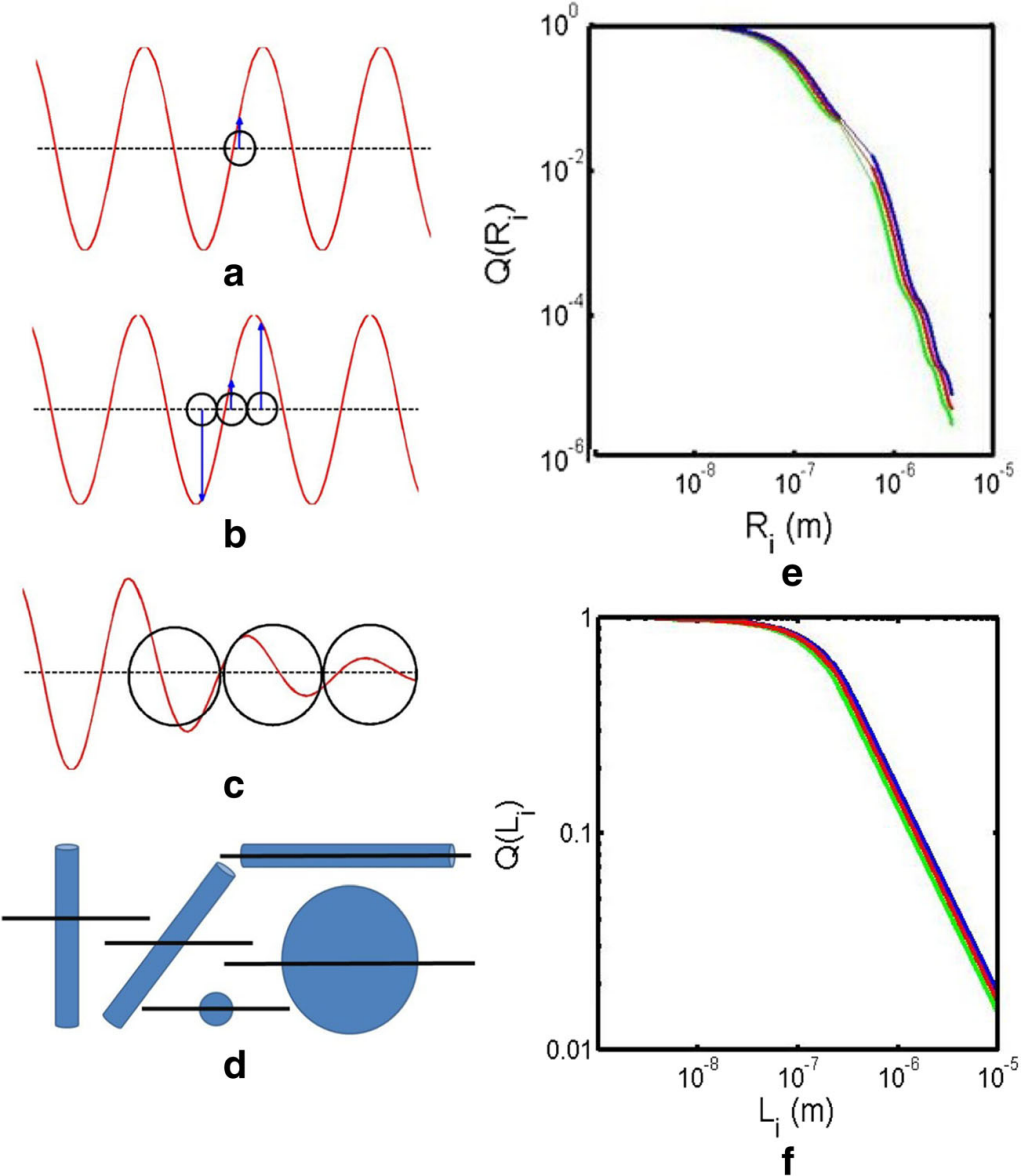
Theoretical treatments of scattering. **a**–**c** Three general scattering regimes were considered by Hall et al. (2016a), namely ** a** Rayleigh limit—where the scattering particle is small in relation to the wavelength of light [<R_i_> < λ/20] (*red line* light wave,* blue arrow* position of the dependent electric field vector). **b** Rayleigh–Gans–Debye limit—where the particle can be reasonably large in relation to the wavelength of light at ∼[0 < <R_i_> < λ/2] such that it produces out-of-phase scattering at different centres of the particle but the light suffers no appreciable loss of intensity as it passes through the particle. **c** Mie scattering regime—where the particle is sufficiently large to both generate out-of-phase scattering and to perturb the intensity of the light as it passes through the aggregate. For the anomalous diffraction approximation of the Mie equation used by Hall et al. (2016a) this description is applicable over the size regime of ∼[2λ < <R_i_> < 15λ]. **d** Schematic highlighting the potential for orientation effects on both the out-of-phase scattering and loss of intensity complications accompanying increasing size and asymmetry of the aggregate. All quantitative descriptions described by Hall et al. (2016a) assume random orientation of the aggregate. **e** Continuous description of the transmittance form factor for a spherical aggregate [Q(R_SPHERE_)] at three different wavelengths (*blue line* 400 nm,* red line* 450 nm,* green line* 500 nm). Interpolation based on a polynomial description of spliced simulations from the three characteristic size regimes is shown in Table 1. **f** Continuous description of the transmittance form factor for rods [Q(L_ROD_)] over a large size regime for three different wavelengths (**a**–**d** adapted, with permission, from Fig. 3 of Hall et al. 2016a;** e**,** f** adapted, with permission, from Fig. 5 of Hall et al. 2016a)

6a $$ i\left(r,\theta \right)=\frac{i_0}{r^2}\left[\frac{9{\pi}^2{\left({V}_i\right)}_{TRACE}^2}{2{\lambda}^4}{\left(\frac{{m_i}^2-1}{{m_i}^2+2}\right)}^2\left(1+{ \cos}^2\theta \right)\right] $$

6b $$ {I}_S=2\pi {r}^2{\displaystyle \underset{0}{\overset{\pi }{\int }}i\left(r,\theta \right) \sin \left(\theta \right)}d\theta $$

6c $$ {I}_0={A}_0{i}_0 $$

6d $$ \frac{I_S}{I_0}=\frac{24{\pi}^3{\left({V}_i\right)}_{TRACE}^2}{\lambda^4}{\left(\frac{{m_i}^2-1}{{m_i}^2+2}\right)}^2\left(\frac{1}{A_o}\right) $$

6e $$ \frac{I_S}{I_0}=L{C}_i\frac{24{\pi}^3{\left({V}_i\right)}_{TRACE}^2}{\lambda^4}{\left(\frac{{m_i}^2-1}{{m_i}^2+2}\right)}^2 $$

In Eq. 6a–e, i_0_ refers to the incident light intensity, A_0_ to the cross-sectional area of illumination of the incident light, L to the optical path length of the transmission measurement and m_i_ to the relative refractive index of the aggregate (relative to the solvent). A numerical value of m_i_ can be calculated (Hall et al. 2016a) on the basis of knowledge of the solvent refractive index, n_0_, the aggregate protein refractive increment, dn/dc_i_, the fractional volume occupation by protein in the trace volume, α_i_, and the partial specific volume, υ (Eq. 7a). The wavelength dependence of the refractive index and refractive increment can be determined using an empirical formula (Perlmann and Longsworth 1948) (Eq. 7b, c).

7a $$ {m}_i=1+{\alpha}_i\left[\left(dn/ dc\right)\left(1/\upsilon \right)\right]/{n}_0 $$

7b $$ n\left(\lambda \right)=1.3403\ \left[0.9922+2.31\times {10}^{-15}/{\lambda}^2\right] $$

7c $$ dn/d{c}_i\left(\lambda \right)=0.19\times {10}^{-3}\left[0.925+2.2\times {10}^{-14}/{\lambda}^2\right] $$

The Rayleigh scattering relationship, shown in Eq. 6a–e, is able to quantitatively account for the scattering of non-polarized light by a compact solute with average radius of less than one-twentieth of the wavelength of light R_i_ < λ/20). In a standard spectrophotometer arrangement (Fig. 4a), the continual encounter of incident light with particles in its path leads to a length-dependent decrease in transmitted light intensity recorded at the detector (Kerker 2013). The description of how the intensity changes with position due to scattering can be formulated via Eq. 8a, in which turbidity, defined as τ, is the first-order decay constant of light intensity, I, with path length, L (Bohren and Huffman 2008; Oster 1955) Rearrangement and integration yields the central section of Eq. 8a, which in turn can be simplified by a series expansion to produce Eq. 8b. When the ratio of scattered light to incident is <0.1, the first term of the series expansion suffices (Eq. 8c) (Kerker 2013).

8a $$ \tau =-\left(\frac{1}{I}\right)\frac{dI}{dL}=-\frac{1}{L}{ \log}_e\left(1-\frac{I_S}{I_0}\right)=2.303(O.D.) $$

8b $$ \tau =\frac{1}{L}{\displaystyle \sum_{n=1}^{\infty}\frac{1}{n}}{\left(\frac{I_S}{I_0}\right)}^n $$

8c $$ \underset{\left( \lim \tau \to 0\right)}{\tau}\approx \frac{1}{L}\left(\frac{I_S}{I_0}\right) $$

Insertion of Eq. 6e into Eq. 8a, b, c yields an expression which accurately describes the path length-corrected turbidity values of small particles at low concentrations, i.e. R_i_ < λ/20, τ_i_ →0, C_i_ → 0).

Although Eq. 8a is capable of predicting the turbidity of small compact particles in the dilute limit, it becomes less suitable as the particles increase either in size, concentration or complexity of their shape^13^ (Bohren and Huffman 2008; Garcia-Lopez and Garcia-Rubio 2008; Garcia-Lopez et al. 2006; Hergert and Wriedt 2012; Kerker 2013). With specific regard to the size and shape of a particle, we note that deviation from the ideal Rayleigh case occurs for two reasons (Fig. 5):

1. Different regions of large aggregates will experience different phases of the incident light’s electric field, thereby producing a complex superposition of the scattered light with a reduction in overall scattering intensity (Bohren and Huffman 2008; Geiduschek and Holtzer 1958) (Fig. 5b).
2. In the case of scattering from very large aggregates, the incident light will be demonstrably reduced in intensity as it travels through the aggregate, compounding the difficulty of accounting for any phase difference produced upon scattering (Elimelech et al. 2013; Kerker 2013) (Fig. 5c, d).

**Fig. 6:**
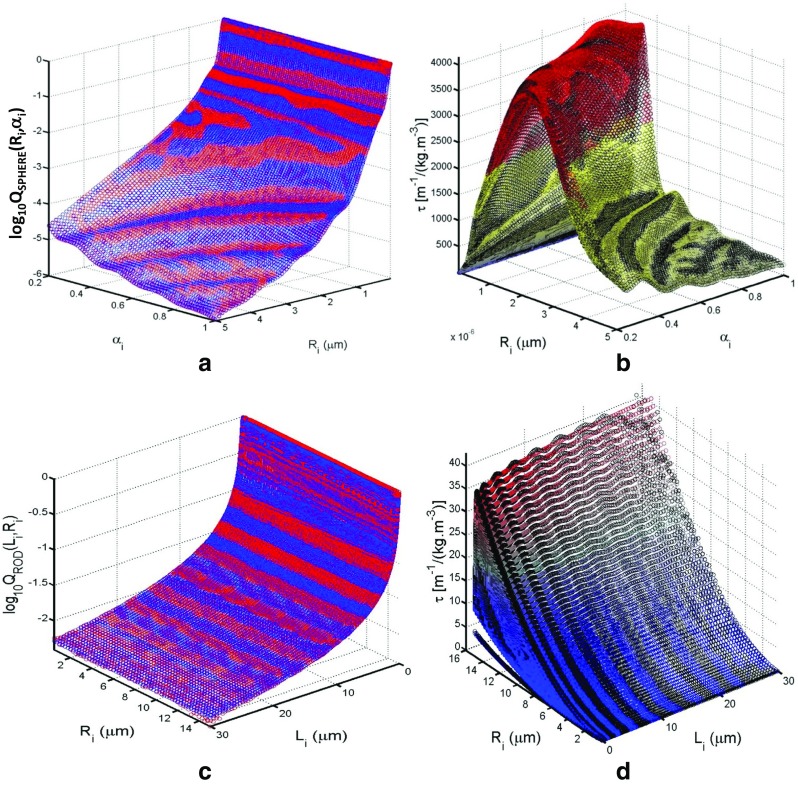
Utilitarian approach developed by Hall et al. (2016a, b) for estimating turbidity. **a** Two-dimensional polynomial fit of simulated Q values for a sphere: fitted values were overlaid onto large sets of the base ten logarithm of Q calculated for a sphere of arbitrary packing fraction α_I_ and radius R_i_, determined using the interpolation technique describe in Fig. 5e (at λ = 400 nm). **b** Specific turbidity (turbidity per kg/m^3^ of aggregate) for a spherical protein aggregate of arbitrary α_i_ and R_i_, calculated using the corresponding value of Q shown in **a**. Protein concentration and mass were respectively set at 1 mg/ml and 5000 g/mole. **c**, **d** Corresponding plots to **a** and **b**, respectively, but this time describing the case for cylindrical rods of arbitrary length and radius. Specific turbidity in **d** was calculated at the same concentration and mass of the protein monomer with a value of the specific fractional volume occupancy of α = 1.0 (adapted, with permission, from Figs. 6, 7, 8 and 9 of Hall et al. 2016a, b)

The Rayleigh–Gans–Debye (RGD) formalism (Debye 1947; Gans 1925; Zimm and Dandliker 1954) is a theoretical approach capable of tackling only the first of these two difficulties and is therefore applicable only to particles of averaged cross-sectional radius, 〈R_i_〉, smaller than λ/{2n_o_(λ)} (Bohren and Huffman 2008). In RGD theory, total scattering intensity is calculated as the sum of the scattering from N discretized centres within the aggregate, on the assumption that the incident light intensity is constant throughout the aggregate (Fig. 6b). A quantity known as the particle form factor P_i_(θ,λ) reflects the degree to which this type of internal interference, generated by effective phase difference, diminishes the scattering recorded for a real particle, i(r,θ)_real_, relative to that measured for an idealized scattering particle (same mass, but point-like dimensions), i(r,θ)_ideal_, such that P_i_(θ,λ) = i(r,θ)_real_/i(r,θ)_ideal_ (Doty and Steiner 1950; Geiduschek and Holtzer 1958) (Table 1). The equivalent term for the transmittance measurement, known as the transmittance form factor, Q_i_, can be directly obtained from P_i_(θ,λ) upon integration to account for all possible orientations of the aggregate in relation to all possible polarizations of the light (Table 1). Within the limits of the approximations inherent in their construction, these form factors can be calculated for any arbitrary shape based on knowledge of the centre-to-centre distances of the discretized scattering centres through use of the Debye equation (Table 1) (Bohren and Huffman 2008; Debye 1947).

**Table 1:** Values of F and Q for the three size regimes and two shape types considered

Approximate size range (for which the description is valid)	Idealized turbidity per unit molecular concentration^a^ (F_i_)	Transmittance form factor^b^ (Q_i_)
Rayleigh 0 ≤ <R_i_> ≤ λ/20	${F}_i=\frac{24\left({M}_1^2{i}^2{\upsilon_i}^2/\left[{\alpha_i}^2{N}_A^2\right]\right)}{\lambda^4}{\left(\frac{m_i^2-1}{m_i^2+2}\right)}^2$	*Q*_*i*_ = 1
Rayleigh-Gans-Debye 0 ≤ <R_i_> ≤ λ/2	${F}_i=\frac{24\left({M}_1^2{i}^2{\upsilon_i}^2/\left[{\alpha_i}^2{N}_A^2\right]\right)}{\lambda^4}{\left(\frac{m_i^2-1}{m_i^2+2}\right)}^2$	${Q}_i={\displaystyle \underset{0}{\overset{\pi }{\int }}P\left(\theta, \lambda \right).\left(1+{ \cos}^2\theta \right). \sin \theta .d\theta }$ ^Where,^ $P\left(\theta, \lambda \right)=\frac{1}{N^2}{\displaystyle \sum_{i=1}^N{\displaystyle \sum_{j=1}^N\frac{ \sin \left(h.{d}_{ij}\right)}{\left(h.{d}_{ij}\right)}}}$ $h=\frac{4\pi n \sin \left(\theta /2\right)}{\lambda }$ ${d}_{ij}=\left|{\overrightarrow{r}}_i-{\overrightarrow{r}}_j\right|$ _For_ *x*_1_ = *R*_*i*_ *n*_*o*_/*λ*_and_ *x*_2_ = *L*_*i*_ *n*_*o*_/*λ* When *x*_2_ < 1; ${Q}_i(sphere)=1-0.955{\left(1-{e}^{-6.48{x}_1}\right)}^{2.40}$ ${Q}_i(rod)={Q}_i(sphere)\left({x}_1\right)\left[1-0.955{\left(1-{e}^{-1.08{x}_2}\right)}^{1.275}\right]$ When *x*_2_ ≥ 1; ${Q}_i(rod)=\frac{{Q_i}_{(sphere)}\left({x}_1\right)}{2.4{x_2}^{0.95}}$
Anomalous diffraction approximation^b^ 2λ ≤ <R_i_> ≤ 15λ	${F}_i=\frac{24\left({M}_1^2{i}^2{\upsilon_i}^2/\left[{\alpha_i}^2{N}_A^2\right]\right)}{\lambda^4}{\left(\frac{m_i^2-1}{m_i^2+2}\right)}^2$	${Q}_i(rod)=\frac{{Q_i}_{(sphere)}\left({x}_1\right)}{2.4{x_2}^{0.95}}$ ${Q}_i(sphere)=\frac{\left[2-\left(\frac{4}{\rho_i}\right) \sin \left({\rho}_i\right)+\left(\frac{4}{{\rho_i}^2}\right)\left(1- \cos \left({\rho}_i\right)\right)\right]}{\left({\left({F}_i\right)}_{RAYLEIGH}/\pi .\left\langle {R_i}^2\right\rangle \right)}$ where, *ρ*_*i*_ = 4*πR*_*i*_(*m*_*i*_ − 1)/*λ*

All expressions are particular for a non-polarized light source and randomly oriented aggregate. All terms are defined in Eq. 3a, b, c; Eq. 4a, b, c; Eq. 6a, b, c, d e; Eq. 7a, b, c; Eq. 9

^a^As defined in Eq. 9 of the text

^b^The anomalous diffraction equation is a good approximation of the Mie scattering description for spheres (Hergert and Wriedt 2012; Kerker 2013; Mie 1908). Arguments have been advanced (Hall et al. 2016a) to suggest that the expression for given for Q_i_(rod) would retain validity in the Mie scattering regime (for a discussion see Cassasa, 1955; Bishop 1989; Buitenhuis et al. 1994; Liu et al. 1998)

An alternative approach to the Debye approximation, developed by Gustav Mie for particles of arbitrary size and shape (Hergert and Wriedt 2012; Mie 1908), accounts for both the decrease in light intensity as it passes through the aggregate and the phase difference in scattered light intensity generated by scattering from widely separated regions of the aggregate molecule (Hergert and Wriedt 2012; Kerker 2013) (Fig. 5c, d). The anomalous diffraction (AD) equation (Table 1) developed by Van de Hulst represents a very accurate simplifying approximation to the Mie scattering equations for aggregates having spherical geometry (Elimelech et al. 2013; van de Hulst 1957). The AD approximation for spheres retains validity over the size regime 2λ ≤ 〈R_i_〉 ≤ 15λ for systems having a relative refractive index, m_i_, of <1.3 (van de Hulst 1957). Importantly, this last requirement represents nearly all conceivable cases of proteins aggregating in standard aqueous buffers. Relatively simple approximate forms of Mie-type solutions for other shapes, such as cylindrical rods, have also been developed and compared to ‘exact’ calculations made using finite element numerical techniques performed over a large range of particle sizes relative to the wavelength of light employed (Bishop 1989; Buitenhuis et al. 1994; Liu et al. 1998) (Table 1).

Based on a recasting of the general turbidity expression into an equation involving three parts, Hall and co-workers (Hall et al. 2016a) laid the foundation for producing an empirical interpolation of the transmittance particle form factor Q_i_ over a wide range of sizes and shapes suitable for describing amyloid growth (Eq. 9) (Fig. 5e, f).

9 $$ \underset{\left( \lim\ \tau \to 0\right)}{\tau_i}={C}_i{F}_i{Q}_i $$

As formulated by Eq. 9, τ_i_, the turbidity at limiting dilution, is composed of three terms, namely C_i_, F_i_ and Q_i_, whereby C_i_ is the scattering particle concentration (units of molecules m^−3^), F_i_ is the idealized turbidity per unit molecular concentration that would be generated if the particle scattered light as a point mass (units: m^2^ molecule^−1^) and Q_i_ is the unit-less transmittance form factor discussed above. Hall and co-workers (Hall et al. 2016a) considered the appropriate functionalization of Eq. 9 for two general shapes, a rod and a sphere of arbitrary internal density, over the three particle size regimes of scattering described in Table 1.

Although values of Q_i_ and F_i_ exist for other shapes (see Bohren and Huffman 2008; Moody et al. 1996), their evaluation from a turbidity signal is problematic, representing, as it does, a type of inverse problem (Hall and Minton 2005; Mroczka and Szczuczynski 2010; Shmakov 2014). Given that a rod and a sphere respectively demonstrate the least and most scattering potential of any regular body, Hall et al. (2016a) suggested that an experimental signal, presumed to reflect amyloid growth, might be empirically decomposed into amyloid (rod-like) and non-amyloid aggregate (assumed spherical) structures. With this basic premise they went on to provide a continuous description of F and Q over a size range spanning the point scattering (R < λ/20) to Mie regime (2λ < R < 15λ) in the form of two-dimensional polynomial interpolants for spheres (Eq. 10a) (Fig. 6a) and rods^14^ (Eq. 10b) (Fig. 6c).

10a $$ { \log}_{10}{Q}_i\left({R}_i,{\alpha}_i\right)={\displaystyle \sum_{j=0}^N{\displaystyle \sum_{k=0}^N{u}_{j,k}{\left({R}_i\right)}^j{\left({\alpha}_i\right)}^k}} $$

10b $$ { \log}_{10}{Q}_i\left({L}_i,{R}_i\right)={\displaystyle \sum_{j=0}^N{\displaystyle \sum_{k=0}^N{w}_{j,k}{\left({L}_i\right)}^j{\left({R}_i\right)}^k}} $$

The coefficients for these two polynomials were evaluated at a series of different wavelengths. With the value of F common to all three different size regimes (Table 1), the differences in turbidity due to shape can be seen to be directly defined by the transmittance particle form factor (Bohren and Huffman 2008; Kerker 2013). Previously calculated numerical examples (Hall et al. 2016a) describing the specific turbidity (i.e. turbidity per set mass of scattering component) produced by a spherical aggregate of arbitrary internal fractional occupancy, and a cylindrical rod for which, α_i_ = 1, are reproduced in Fig. 6b, d. In the case of multiple aggregate species, the turbidity for a solution of aggregates of different sizes and shapes can be calculated as the sum of the contributions to turbidity from each particle (Eq. 11) (Bohren and Huffman 2008; Kerker 2013).

11 $$ \underset{\left( \lim\ \tau \to 0\right)}{\tau }={\displaystyle \sum_{i=1}^N{\tau}_i} $$

The aim of this review is to consider the kinetics of aggregate formation as monitored by the turbidity assay. In the section Consensus kinetic models of aggregate growth, we review different consensus kinetic behaviours of amyloid/aggregate formation, summarizing them into a set of limited basis models (Table 2). Together with the geometric and turbidimetric transforms reviewed in the previous sections, these consensus kinetic models are used to simulate characteristic turbidity signatures associated with particular mechanistic sub-types (Ghosh et al. 2010; Hall and Edskes 2012; Kashchiev 2015).

## (iii) Consensus kinetic models of aggregate growth

An extremely general description of non-specific cluster formation was made over 150 years ago by von Smoluchowski (1916, 1917). In that approach, for a single isomeric state^15^, the rate of formation of an aggregate species is given by the total balance of all possible formation and breakage events (Eq. 12a, b),

12a $$ {\left(\frac{d{C}_k}{dt}\right)}_{growth}={\displaystyle \sum_{i=1}^{k-1}{f}_{i,k-i}{C}_i{C}_{k-i}}+{\displaystyle \sum_{j=k+1}^zb{}_{k,j-k}C_j} $$

12b $$ {\left(\frac{d{C}_k}{dt}\right)}_{loss}=-{\displaystyle \sum_{i=1}^z{f}_{k,i}{C}_k{C}_i}-{\displaystyle \sum_{j=1}^{k-1}b{}_{j,k-j}C_k} $$

**Fig. 7:**
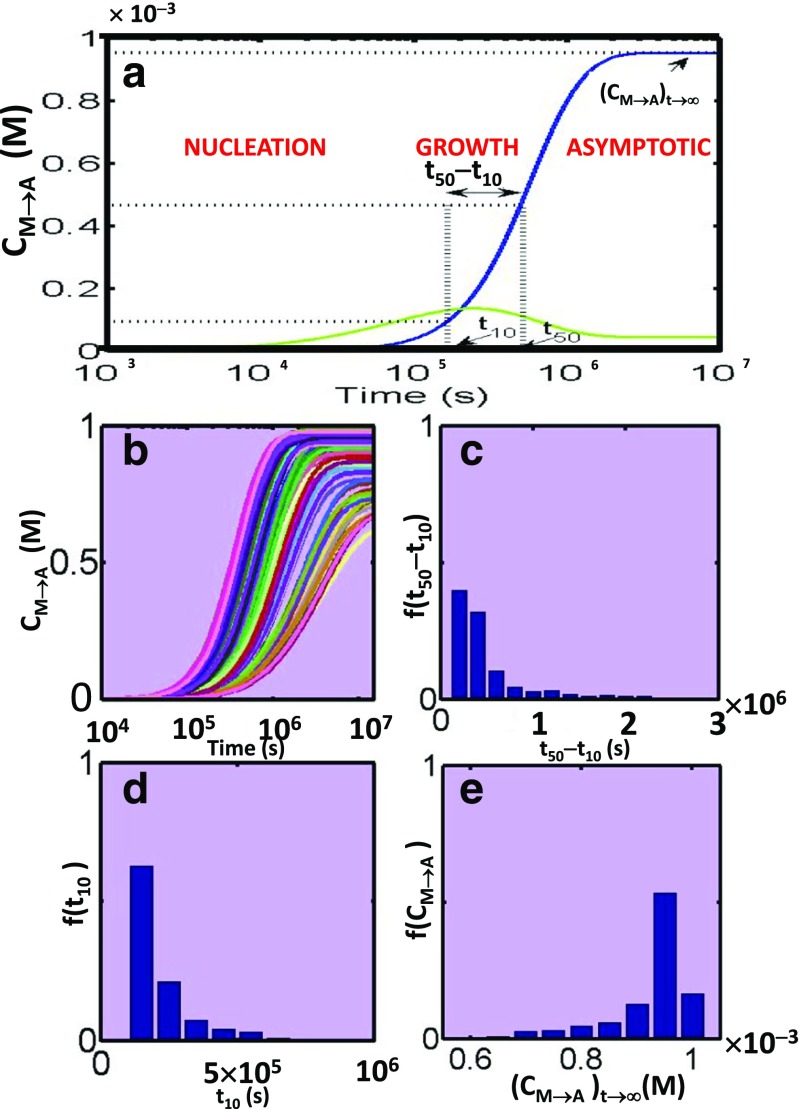
Schematic of amyloid kinetics. **a** Characteristic features of amyloid nucleation–growth polymerization kinetics include a characteristic lag/nucleation phase, a steep growth phase and an asymptotic endpoint. A simple scheme for reducing the data to parameters reflecting each of these characteristic features is included. These parameters include (1) the kinetic tenth time, *t*_*10*_ (time to reach 10 % of reaction), reflecting the nucleation phase, (2) a composite term reflecting the difference between half-time, *t*_*50*_, and kinetic tenth time (*t*_*50*_ *− t*_*10*_) characteristic of the growth phase and (3) the time-independent value of the extent of the monomer incorporated into amyloid, (*C*_*M→A*_)_*t→∞*_, characterizing the asymptotic phase.* Blue line* Value of C_M→A_ as a function of time,* green line* the corresponding concentration of monomer as critical nucleus (nC_N_) as a function of time (adapted, with permission, from Fig. 1d of Hall et al. 2016a, b). **b** Data reduction and analysis. In the case of drug screening for amyloid inhibitors, replicate measurements of the measured growth kinetics are decomposed into a set of characteristic values (such as the set of parameters described in Fig. 7a), with resultant values represented as a fractional histogram. **c**–**e** Fractional histogram representation of the surrogate markers of the nucleation (**d**), growth (**c**) and asymptotic (**e**) regions derived from the simulations shown in** b** (adapted, with permission from Fig. 2 of Hall et al. 2016b), with simulated results multiplied by a constant value to more closely reflect the time course and concentration profiles shown in subsequent cases)

Within this cluster growth formalism, the concentration of an aggregate composed of k monomers is denoted by C_k_. The chemical rate constant^16^ for formation of a species of size k from two smaller species j and k−j is denoted by f_j,k-j_. Similarly, the chemical rate constant describing the breakage of a species of size k into two species, j and k−j, is denoted by b_k−j,j_. With an appropriate choice of rate constants, the Smoluchowski cluster/condensation rate model can be used to describe aggregation processes of great complexity (Aldous 1999). However, despite this potential for diversity, the kinetics of amyloid formation have repeatedly been shown to comport to a subset of the possible model space, defined by Eq. 12a, b, with this subset known as nucleated growth^17^ (Jarrett and Lansbury 1992; Lomakin et al. 1996; Masel et al. 1999; Wetzel 2006) (Fig. 7). In its most general form this mode of aggregation involves the introduction, or slow production, of a structural nucleus within a pool of monomeric proteins^18^ which are themselves capable of joining to the nucleus and adopting the template structure encoded by it (Jarrett and Lansbury 1992; Petkova et al. 2005; Wetzel 2006). As the amyloid reaction proceeds, fibres can break apart (Hall and Edskes 2009, 2012, 2004; Xue et al. 2008) or join together (Pallitto and Murphy 2001; Binger et al. 2008, Michaels and Knowles 2014). Joining of fibres may occur in either an end-to-end fashion (Binger et al. 2008), a lateral side-to-side manner (Pallitto and Murphy 2001; Nichols et al. 2002; Kanno et al. 2005) or by a random process (Mishra et al. 2011) to produce a low-density matrix. Irrespective of their exact form, idealized nucleation–growth models typically display sigmoidal-type association kinetics if the nucleation step is allowed to occur spontaneously (Jarrett and Lansbury 1992), or exponential-type association kinetics without a lag-phase, if nucleation is bypassed by seeding the system with template (Paravastu et al. 2009; Wetzel 2006). In accordance with Fig. 7, the kinetics can be parameterized with constants describing the nucleation, growth and asymptotic stages of the reaction (Hall et al. 2016b). Alternatively, the kinetic traces may be fitted to equations derived from mechanistic models of amyloid growth, to yield the most parsimonious set of rate constant parameters (Pallitto and Murphy, 2001; Morris et al. 2009; Smith et al. 2006).

In the following subsections we discuss a number of potential variants in the nucleated growth model, summarizing the basic kinetic behaviour with an appropriate chemical rate equation (Table 2). In the formulation and discussion of kinetic data reflecting the various limiting cases of aggregation behavior, we make two assumptions:

1. Polymer distributions are approximated by their weight average. A more formal description of this approximation for the weight average degree of polymerization, is shown by Eq. 13:

**Table 2:**
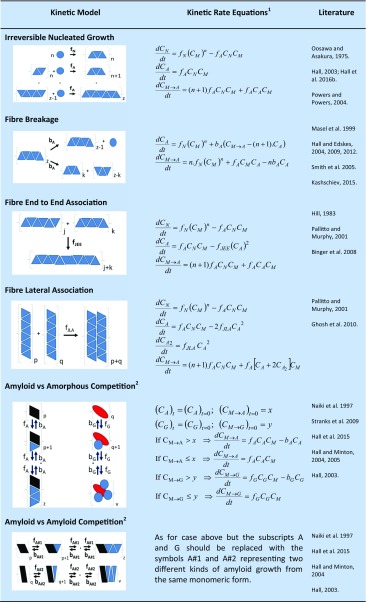
Kinetic equations for six different cases of amyloid aggregation

^1^ Monomer was calculated via conservation of mass arguments with terms as appropriate

^2^ Seeds are regarded as fixed i.e. non-dissociable

13 $$ \left\langle i\right\rangle =\frac{{\displaystyle \sum_{i=2}^z{C}_i.{i}^2}}{{\displaystyle \sum_{i=2}^z{C}_i.i}} $$.

(2) In discussing either breakage, competitive growth or fibre joining, a separation of time scales for the monomer/polymer mass and polymer mass/polymer distribution time scales will often be invoked (Bridstrup and Yuan 2016; Hall and Minton 2004). A more formal statement of this conceptual tool is given by the following mechanistic approximation [Eq. 14]:

14 $$ monomer\begin{array}{c}\hfill \overset{fast}{\to}\hfill \\ {}\hfill \leftarrow \hfill \end{array} polymer\  mass\begin{array}{c}\hfill \overset{\mathrm{fast}\ \mathrm{or}\ \mathrm{slow}}{\to}\hfill \\ {}\hfill \leftarrow \hfill \end{array} polymer\  distribution $$

With regard to the first assumption (described in Eq. 13), we note that a few researchers (Arosio et al. 2012; Ghosh et al. 2010; Hall and Edskes 2004, 2009; Hall et al. 2015) have developed methods for simulating amyloid kinetics that yield full distribution information as a function of time. Although these methods are more informative than the approximation adopted by Eq. 13, they are also necessarily more complex. Due to the focus of this review being on the transformation of the distribution by turbidimetric assay procedures, we have opted to make a trade-off: a level of exactness for ease of discourse^19^. In the cases where no literature-derived chemical rate equation exists (or alternatively no literature derivation possessing a relatively transparent formulation exists), we have cited the relevant literature but put forth an approximate relation.

Of all possible permutations available to Eq. 12a, b, the following six limiting cases of templated growth are regarded as having principal importance in this review:

- Irreversible nucleation–growth model
- Nucleation growth with fibre breakage
- Fibre end-to-end association
- Fibre lateral association
- Amyloid versus amorphous competition
- Amyloid versus amyloid competition

**Fig. 8:**
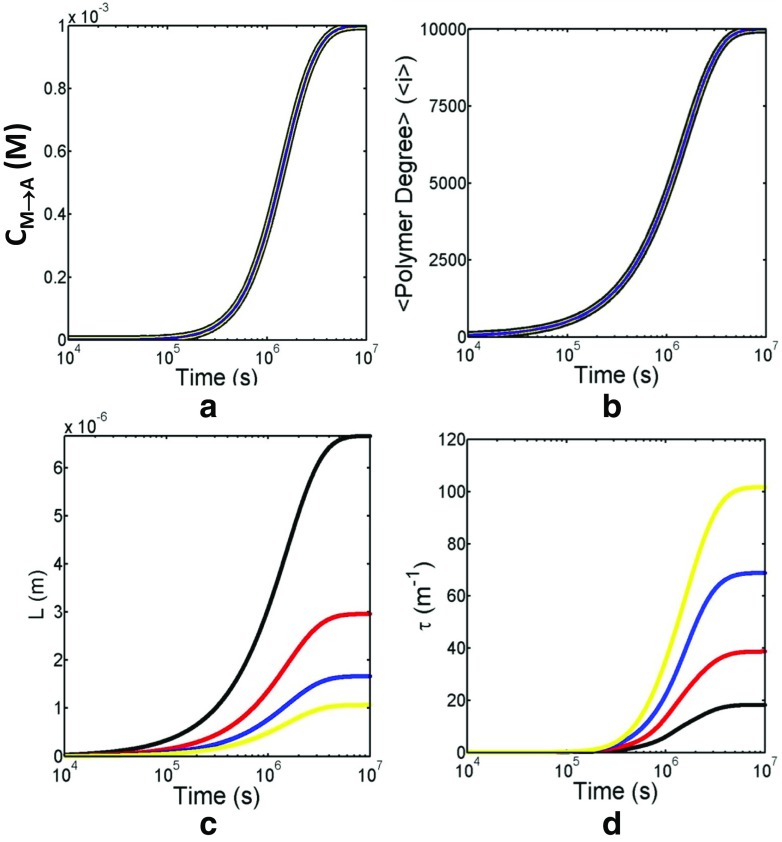
Irreversible nucleation–growth model—effect of fibre width on the turbidity transform. Simulation of four cases of irreversible amyloid growth which, although exhibiting identical growth kinetics, differ in the radius of the amyloid fibre produced, such that R_A_ = 4 nm (*black line*), 6 nm (*red line*), 8 nm (*blue line*) or 10 nm (*yellow line*). **a** Concentration of monomer incorporated into amyloid, C_M→A_, as a function of time for four different cases of amyloid radius (*single line* for all four cases reflects identical growth kinetics dictated by imposition of identical rate constants. **b** Average polymer degree (*<i>*) of aggregate as a function of time for the four different cases of amyloid fibre radius (*single line* for all four cases is due to identical nucleation and growth kinetics brought about by use of identical rate constants). (**c**) Length (*L*) of amyloid fibres as a function of time for the four different cases of amyloid fibre radius. As per volume conservation requirements, fibres of different width lengthen in a manner proportional to L_1_/L_2_ = (R_ROD2_)^2^/(R_ROD1_)^2^. **d** Turbidity (*τ*) of amyloid fibres as a function of time for the four different cases of amyloid fibre radius calculated using the transforms shown in Eqs. 9, 10a, b and 11. For the same average degree of polymerization, wider fibres of shorter length exhibit much greater turbidity than narrow fibres of longer length. Common parameters: f_A_ =10 M^−1 ^s^−1^, f_N_ = 1 × 10^−7^ M^−1 ^s^−1^, b_A_ = 0 s^−1^, n = 2, (C_M_)_tot_ = 1 × 10^−3 ^M, R_1_ = 2 nm, M_1_ = 27.65 kg/mole, υ = 0.73 × 10^−3^ m^3 ^kg^−1^, α = 1.0

**Fig. 9:**
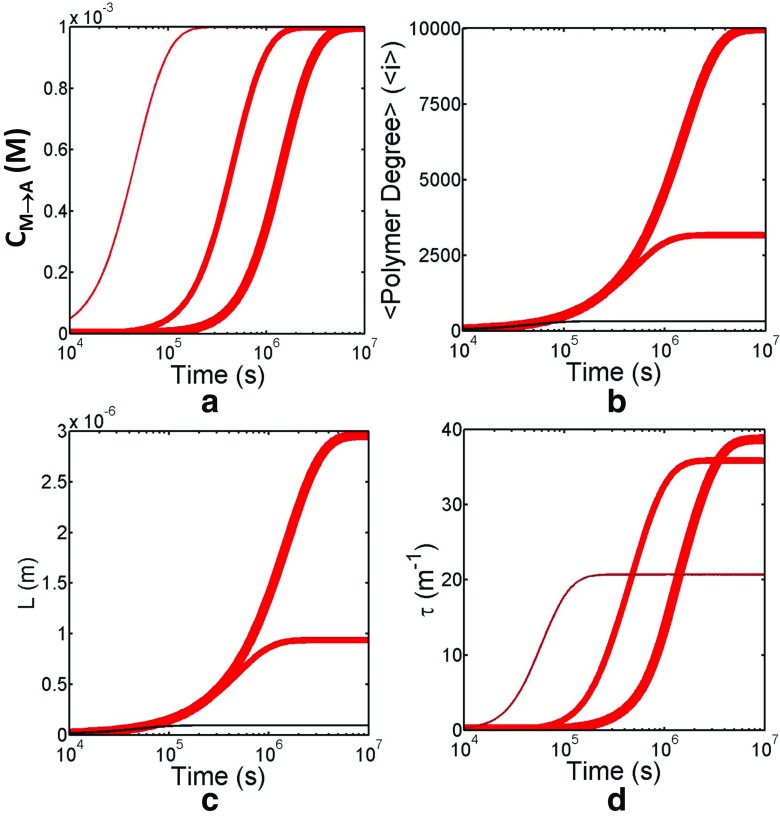
Irreversible growth model—effect of nucleation rate on the turbidity transform. Simulation of three cases of irreversible amyloid growth which, although rod widths are identical, differ in the rate of nucleation of amyloid fibre produced such that f_N_ = 1 × 10^−7 ^M^−1 ^s^−1^ (*thick red line*), f_N_ = 1 × 10^−6^ M^−1^ s^−1^ (*intermediate-thick red line*) and f_N_ = 1 × 10^−4^ M^−1^ s^−1^ (*thin line*). **a** Concentration of monomer incorporated into amyloid (*C*_*M→A*_) as a function of time for the three different cases of amyloid nucleation rate. Faster nucleation rates dictate faster growth kinetics due to a greater number of extendable nuclei being formed. **b** Average polymer degree (*<i>*) of aggregate as a function of time for three different cases of amyloid fibre nucleation. Slower nucleation rates lead to larger average degrees of polymerization. **c** Length (*L*) of amyloid fibres as a function of time for the three different cases of amyloid fibre nucleation rate. As per the average degree of polymerization, for fixed fibre geometry, slower nucleation rates lead to longer fibres. **d** Turbidity (*τ*) of amyloid fibres as a function of time for the three different cases of amyloid fibre nucleation rate. As can be noted from Fig. 6d, the specific turbidity becomes relatively insensitive to length after the fibres are longer than ∼2λ. In practice this finding means that for conditions producing very small fibre distributions, due to rapid nucleation kinetics, the measured turbidity value reflecting the asymptotic limit will be lower than that obtained for a system producing the same mass concentration of amyloid using slower nucleation kinetics. Common parameters: f_A_ = 10 M^−1 ^s^−1^, f_N_ = 1 × 10^−7 ^M^−1^ s^−1^, b_A_ = 0 s^−1^,* n*= 2, (C_M_)_tot_ = 1 × 10^−3 ^M, R_1_ = 2 nm, M_1_ = 27.65 kg/mole, υ = 0.73 × 10^−3^ m^3 ^kg^−1^. R_A_ = 6 nm, α = 1.0

**Fig. 10:**
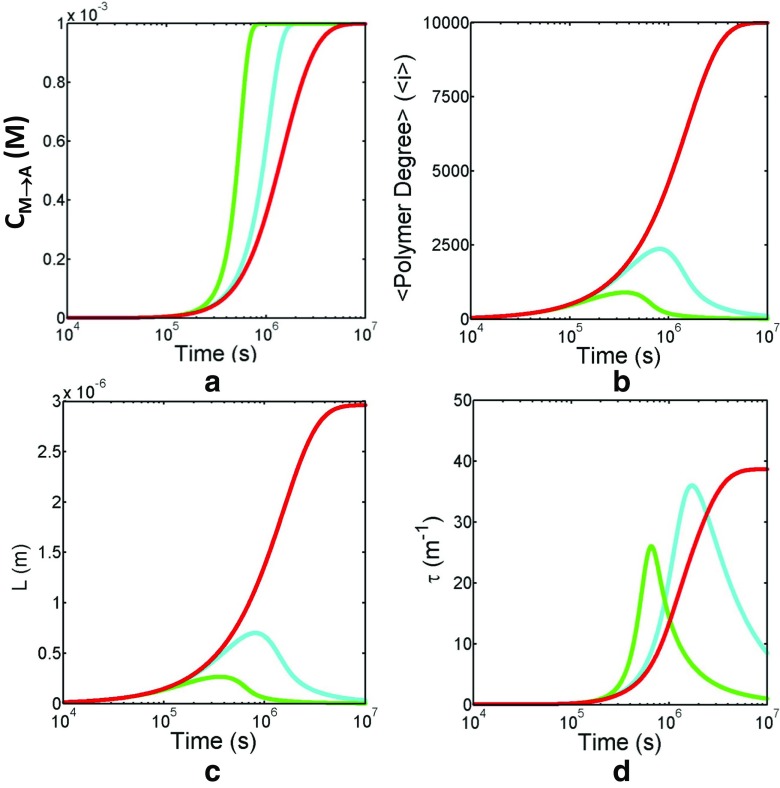
Reversible growth model—effect of breakage rate on the turbidity transform. Simulation of three cases of reversible growth with breakage, in which the fibre width is the same for all cases, but the fibres differ in their intrinsic tendency towards breakage (or as some have termed ‘frangible’) such that b_A_ = 0 s^−1^ (*red line*), b_A_ = 1 × 10^−9^ s^−1^ (*cyan line*) and b_A_ = 1 × 10^−8^ s^−1^ (*green line*). **a** Concentration of monomer incorporated into amyloid (*C*_*M→A*_) as a function of time for the three different cases of intrinsic breakage rate. Note that faster breakage rates lead to an effective reduction in both the nucleation and growth phases with a subsequent faster attainment of the asymptotic value. **b** The average polymer degree of aggregate (*<i>*) as a function of time for the three different cases of intrinsic breakage rate. Slow breakage rates, relative to the rate of attainment of the polymer mass equilibrium, can lead to a slow reduction in the average polymer degree in a manner effectively temporally decoupled from the time scale of attainment of the monomer/polymer mass equilibrium (see Eq. 14). **c** Length (*L*) of amyloid fibres as a function of time for the three different cases of breakage rate. As for the just described case of <i> vs. t, slow intrinsic breakage rates can lead to an uncoupling between the times scales of the total mass of protein existing as amyloid and the production of shorter fibre distributions from longer initial distributions. **d** Turbidity (*τ*) of amyloid fibres as a function of time for three different cases of amyloid breakage rate. As the fibres shorten below the ∼2λ length limit the turbidity decreases significantly, even though there is noeffective decrease in C_M→A_. Common parameters: f_A_ = 10 M^−1 ^s^−1^, f_N_ = 1 × 10^−7^ M^−1 ^s^−1^, n = 2, (C_M_)_tot_ = 1 × 10^−3 ^M, R_1_ = 2 nm, M_1_ = 27.65 kg/mol, υ = 0.73 × 10^−3^ m^3 ^kg^−1^. R_A_ = 6 nm, α = 1.0

**Fig. 11:**
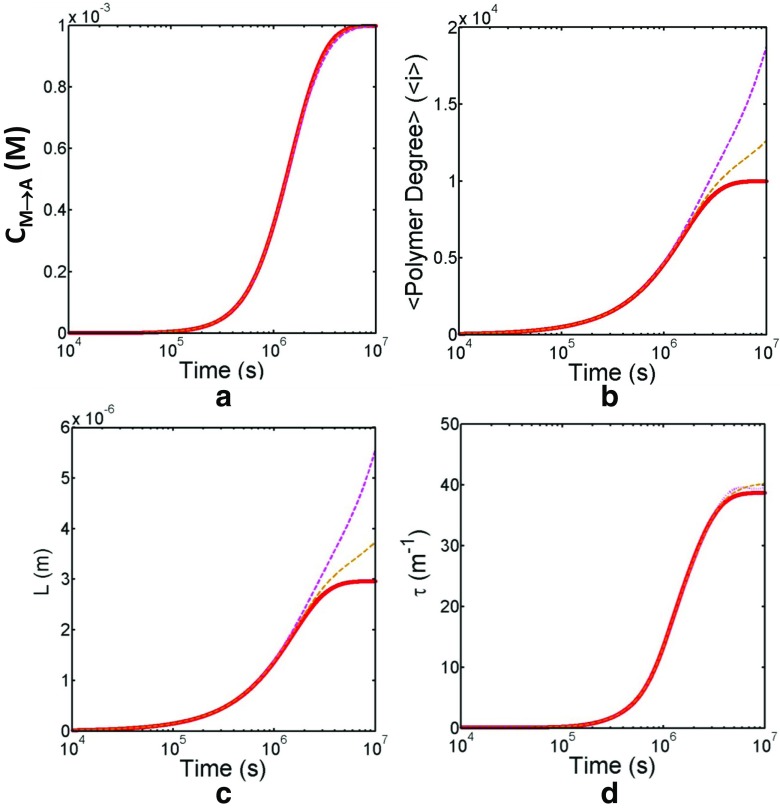
Fibre end-to-end joining model—effect of association rate on the turbidity transform. Simulation of three cases of the fibre-joining model in which the amyloid fibre width is kept constant but the fibre joining rate (f_JEE_) is set at f_JEE_ = 0 M^−1^ s^−1^ (*solid red line*), f_JEE_ = 0.3 M^−1^ s^−1^ (*dashed orange line*) and f_JEE_ = 1.0 M^−1^ s^−1^ (*dashed magenta line*). **a** Concentration of monomer incorporated into amyloid (*C*_*M→A*_) as a function of time for the three different cases of joining rate considered. The relatively low numerical values used for the joining rate constants in these simulations mean that the polymer redistribution kinetics are effectively decoupled from the monomer/polymer mass kinetics (see Eq. 14). As such, no change in the kinetics of monomer incorporation is observed in the three different cases considered. **b** Effect of fibre-joining rate on the average polymer degree (*<i>*) as a function of time. Faster rates of increase in polymer degree are affected by faster joining rates, but this occurs slowly in the present case due to the relatively low values of f_JEE_ specified. **c** Length (*L*) of amyloid fbres as a function of time for the three different cases of fibre-joining rate considered. Note that the fibres slowly lengthen under the regime of joining rate constants selected. **d** Turbidity (*τ*) of amyloid fibres as a function of time for the three different cases of fibre-joining rate considered. No change in turbidity is detectable amongst the three cases of fibre-joining rate considered. This result follows from relations summarized in Table 1 (represented pictorially in Fig. 6d) whereby an increase in length, at constant polymer mass concentration, should be largely invisible to detection by turbidity. Common parameters: f_A_ = 10 M^−1^ s^−1^, f_N_ = 1 × 10^−7 ^M^−1^ s^−1^, n = 2, (C_M_)_tot_ = 1 × 10^−3 ^M, R_1_ = 2 nm, M_1_ = 27.65 kg/mol, υ = 0.73 × 10^−3^ m^3 ^kg^−1^. R_A_ = 6 nm, α = 1.0

**Fig. 12:**
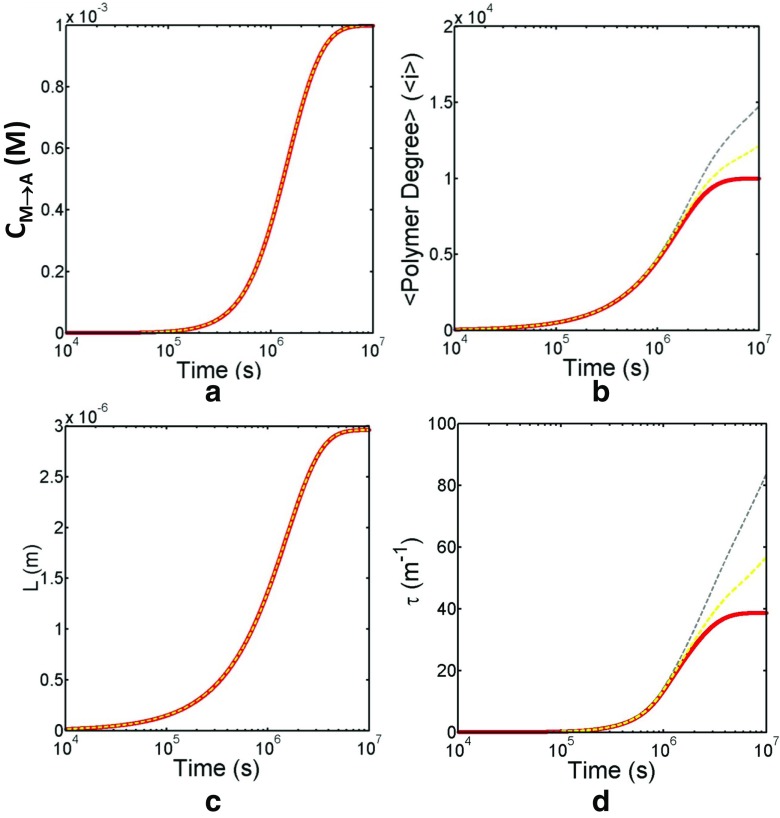
Fibre lateral association model—effect of lateral association rate on the turbidity transform. Simulation of the fibre lateral association model in which fibres are able to form laterally-associated ‘mature’ fibres consisting of fibre dimers, for three cases of the joining lateral association rate constant (f_J__LA_) are explored, with f_J LA_ = 0 M^−1 ^s^−1^ (*solid red line*), f_J LA_ = 0.3 M^−1^ s^−1^ (*dashed yellow line*) and f_J LA_ = 10 M^−1^ s^−1^ (*dashed grey line*). **a** Concentration of monomer incorporated into amyloid (*C*_*M→A*_) as a function of time. All three simulated cases of different intrinsic lateral association rate overlap as the fibre-joining rate is assumed not to influence the reactivity of the individual fibre ends. **b** Average polymer degree (*<i>*) as a function of time. The low numerical values selected for the fibre lateral association rate constants mean that the asymptotic limit of the average polymer degree is approached very slowly. **c** Simulated length (*L*) of amyloid as a function of time for the three examined cases of fibre lateral association rate. The coincident behavior is a consequence of the two simplifying assumptions that fibre size distributions are approximated by their average, <i>, and that fibre lateral association occurs at the fibre midpoint (see text on this point for a discussion). **d** Simulated turbidity (*τ*) of amyloid solution as a function of time for the three cases of fibre lateral association rate. Attainment of an asymptotic limit in the turbidity profile is delayed (or not apparent) for the cases of faster lateral association rate. Note that based on relations presented in Table 1 and Fig. 6d, a change in fibre width, at constant aggregate mass concentration, will result in an increase in turbidity. Common parameters: f_A_ = 10 M^−1 ^s^−1^, f_N_ = 1 × 10^−7 ^M^−1 ^s^−1^, n = 2, (C_M_)_tot_ = 1 × 10^−3 ^M, R_1_ = 2 nm, M_1_ = 27.65 kg/mol, υ = 0.73 × 10^−3^ m^3^ kg^−1^. R_A_ = 6 nm, α = 1.0

**Fig. 13:**
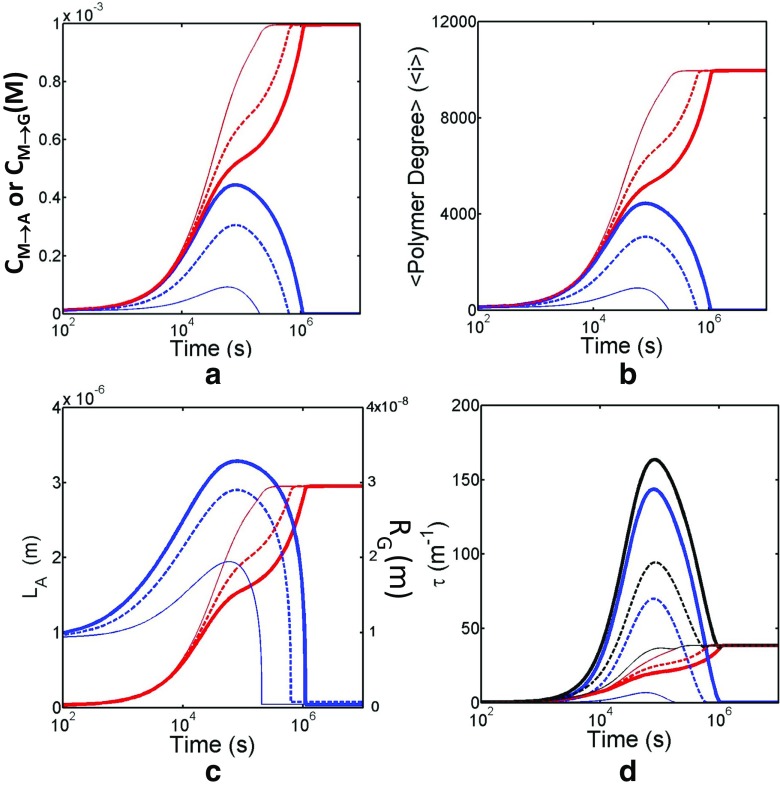
Amyloid vs. amorphous competition—effect of relative rates of amorphous and amyloid growth on the turbidity transform. Simulation of three cases of competitive reversible-seeded growth in which the rate constants reflecting amyloid growth are kept constant but the amorphous growth kinetics are modified by varying the amorphous aggregate association constant (f_G_), such that f_G_ = 50 M^−1 ^s^−1^ (*thin solid lines*), f_G_ = 150 M^−1^ s^−1^ (*intermediate-thick dashed lines*) and f_G_ = 250 M^−1^s^−1^ (*thick solid lines*) whereby the* red* version of the particular line type refers to the amyloid species and the* blue* version of the line refers to the amorphous species. **a** Concentration of monomer in amyloid (*C*_*M→A*_) or amorphous aggregate (*C*_*M→ G*_) as a function of time for the three different cases of amorphous relative to amyloid growth. In all cases the choice of rate constants ensures that the amyloid is ultimately more thermodynamically stable than the amorphous aggregate. Relatively fast amorphous association rates lead to a significant extent of monomer being initially converted into the amorphous form, prior to its eventual dissociation and re-incorporation into the amyloid state. **b** Average polymer degree of amyloid (*<i*_*A*_ *>*) and amorphous (*<i*_*G*_ *>*) as a function of time for the different simulated cases of relative rates of amorphous to amyloid growth. Due to the fact that the simulation model specifies seeded growth (in which the number concentration of amyloid and amorphous species are fixed at constant values throughout—see Table 1), <i_A_> (*red lines*) attains the same eventual value for all cases of relative growth. Similarly, the average degree of polymerization of the amorphous aggregate, <i_G_> (*blue lines*) approaches a value close to the starting value of the amorphous seed, <i_G_>_t=0_, in all cases. **c** Average size of aggregate species as a function of time for three simulated cases of relative rates of amorphous vs. amyloid growth, with the* left y-axis* specifying the length (*L*_*A*_) of the amyloid species and the* right y-axis* describing the radius (*R*_*G*_) of the amorphous aggregate species. The faster cases of amorphous growth lead to aggregates of larger radius (compare ∼32 to 20 nm) whereas L_A_ never surpasses its maximum value due to a slow approach to equilibrium from below (i.e. no overshoot is seen). **d** Turbidity (*τ*) as a function of time for the three cases reflecting different relative rates of amorphous to amyloid growth. * Coloured lines* Component turbidity generated by the amyloid (*red line*) and amorphous (*blue line*) species.* Black lines* represent the total resultant turbidity.* Line style* is dictated by the different cases reflecting the rate of amorphous to amyloid growth:* solid thick lines* relatively fast amorphous growth,* dashed intermediate-thick lines* amorphous growth,* thin solid lines* slow amorphous growth. Common parameters: f_A_ = 250 M^−1^ s^−1^, b_A_ = 1 × 10^−3 ^s^−1^, b_G_ = 1 × 10^−2 ^s^−1^, (C_M_)_tot_ = 1 × 10^−3 ^M, R_1_ = 2 nm, M_1_ = 27.65 kg/mol, υ = 0.73 × 10^−3^ m^3 ^kg^−1^. R_A_ = 6 nm, α_A_ = α_G_ = 1.0, (C_A_)_t=0_ = 1 × 10^−7^M, (C_G_)_t=0_ = 1 × 10^−7^M, <i_A_>_t=0_ = 100, <i_G_>_t=0_ = 100

**Fig. 14:**
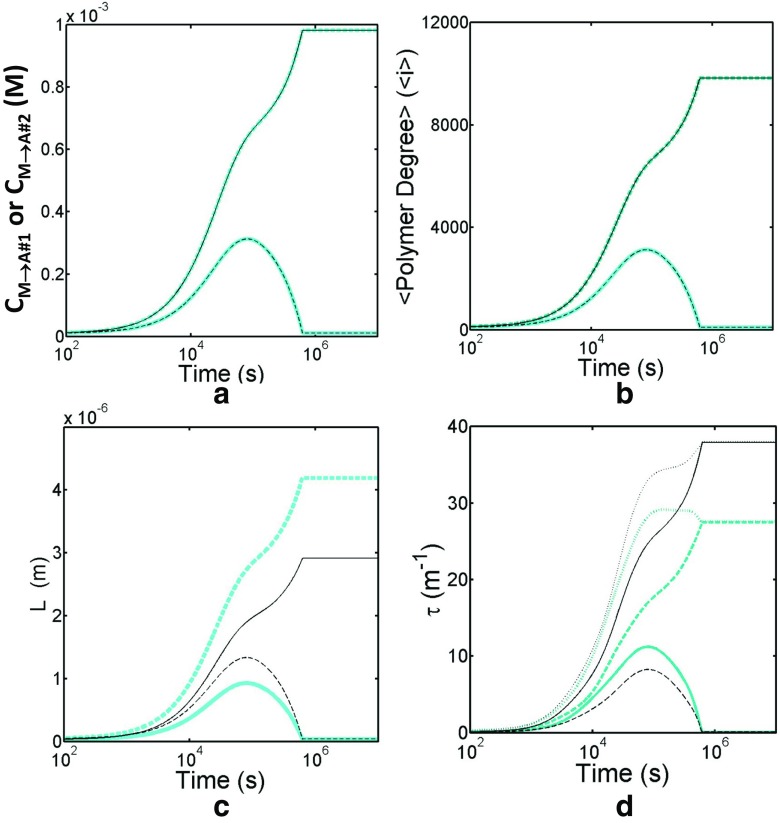
Amyloid vs. amyloid competition—effect of relative rates of growth between two geometric forms of amyloid on the turbidity transform. Simulations showing two cases of competitive reversible seeded growth between two amyloid types possessing quite subtle differences in geometry such that type #1 fibres have a radius R_A#1_ of 5 nm (*dashed lines*) and type #2 fibres have a radius of R_A#2_ = 6nm (*solid lines*). Two cases of reversible growth are produced by swapping the sets of kinetic rate constants. The simulation showing eventual more stable growth of the narrow type #1 fibres in the thermodynamic limit is defined by Case A (*cyan lines*; f_A#1_ = 150 M^−1 ^s^−1^, b_A#1_ = 0.001 s^−1^, f_A#2_ = 250 M^−1 ^s^−1^, b_A#2_ = 0.01 s^−1^). The simulation ultimately reflecting more stable growth of the thicker type #2 fibres is defined by Case B (*black lines*; f_A#1_ = 250 M^−1 ^s^−1^, b_A#1_ = 0.01 s^−1^, f_A#2_ = 150 M^−1 ^s^−1^, b_A#2_ = 0.001 s^−1^). **a** Concentration of monomer incorporated into either of the two types of amyloid (*C*_*M→A#1*_ *or C*_*M→ A#2*_) as a function of time. As the kinetics are simply reversed between the two different cases, Case A (*cyan lines*) and Case B (*black lines*) are coincident. **b** Average polymer degree (*<i>*) reflecting either type #1 amyloid (*<i*_*A#1*_ *>*) or type #2 amyloid (*<i*_*A#2*_ *>*) as a function of time. As the polymer degree per se is insensitive to the geometry of the amyloid, these two cases are also coincident (being simple reversals of the kinetic rate constants). **c** Simulated length (*L*) of amyloid as a function of time for Case A (*cyan lines*), whereby the thin fibre (*dashed lines*) is eventually dominant, and Case B (*black lines*) for which the thick fibre (*solid line*) is eventually dominant. The differences in width between the two fibre types means that different lengths are produced between the two cases even though the average degree of polymerization is identical. **d** Turbidity (*τ*) of amyloid solution as a function of time for two cases of competitive amyloid growth. The resultant turbidity for both cases is shown by* dashed lines* (*thin black dashed line* Case B,* thick cyan dashed line* Case A). Note the unusual kinetics (different to the ideal type shown in Fig. 7a) produced by very minor differences in fibre geometry. Common parameters: (C_M_)_tot_ = 1 × 10^−3^ M, R_1_ = 2 nm, M_1_ = 27.65 kg/mol, υ = 0.73 × 10^−3^ m^3^ kg^−1^. α_A#1_ = 1.0, α_A#2_ = 1.0

Functional kinetic models for each of these limiting cases have been provided (Table 2). Rate models are presented in differential equation format due to the straightforward manner in which ordinary differential equations (ODE) can be related to mechanism by inspection^20^. Figs. 8, 9, 10, 11, 12, 13, and 14 show the resultant chemical kinetics and turbidity transformation for each case. We discuss both the kinetic behavior and the turbidity transformation (effected through application of Eqs. 9–11 to the simulated chemical data) to each case in turn.

### Irreversible nucleation-growth

 Oosawa and colleagues developed the first nucleation–growth-type kinetic models to describe the polymerization of helical fibers formed by the cytoskeletal protein actin (Oosawa and Asakura 1975; Oosawa and Kasai 1962). Despite potential structural and mechanistic differences^21^, others have adapted these Oosawa class of kinetic models to empirically describe the time-course of amyloid formation (e.g. Morris et al. 2009). To effect simulation of irreversible nucleation–growth kinetics, all fibre breakage rate constants in Eq. 12a, b are set equal to zero (i.e. all b_i, j_ = 0). From the time of the original work by von Smoluchowski (von Smoluchowski 1917, 1916), many have attempted a first principles estimation of association rate constants (f_i, j_) based on the component characteristics (e.g. Hall et al. 2005; Hill 1983; Pallitto and Murphy 2001). On the assumption that amyloid growth occurs primarily via monomer addition, Hall and Hirota (2009) calculated a numerical value for all f_1,j_ association constants (based on hydrodynamic reasoning) and then used these parameter values to perform a full distribution simulation of amyloid growth, exploring effects of peptide position and role of peptide flanking sections. As a further simplification, in the Oosawa-type models all forward association rate constants are assigned one of two different values depending on their positional relation to the polymerization event featuring the nucleus, considered as possessing a size, n (Masel et al. 1999; Oosawa and Asakura 1975). In the Oosawa approximation, association rate constants (f_i,j_) are set equal to either f_N_, denoted as the nucleation rate constant for species i+j ≤ n, or f_A_, termed the growth rate constant for association of species i,j where i+j > n. Kinetics comporting to the nucleation–growth scheme are generated by calculating the rate of formation and loss of each species on the condition that f_A_ >> f_N_ (Arosio et al. 2012; Hall 2003). A group of three coupled ODEs (shown in Table 2) representing the Oosawa–Kasai–Asakura approximation (Hall 2003; Oosawa and Asakura 1975) is produced upon appropriate summation of the complete set of ODEs specifying the rate of formation and loss of each aggregate species (Hall et al. 2015, 2016a, 2016b). Within this reduced set of equations the nucleus number concentration is given by C_N_ and the sum of the number concentrations of all amyloid fibre species is described by C_A_ (whereby C_A_ = ΣC_i_ from n+1 to the maximum amyloid degree). The number concentration of all monomers within amyloid form is denoted as C_M→A_ (whereupon C_M→A_ = Σi. C_i_ from n+1 to the maximum amyloid degree). On the basis that the signal measure of amyloid formation reflects C_M→A_, methods have been proposed for deducing the nucleus size and the nucleation and growth rate constants from logarithmic transform plots (Hall 2003; O’Nuallain et al. 2006; Oosawa and Asakura 1975; Powers and Powers 2006). A noted feature of the irreversible nucleated growth mechanism is that, dependent upon the relative rates of nucleation versus growth, a demonstrable amount of monomer existing as nucleus species can be present at the reaction end (e.g. see Fig. 7) (Hall et al. 2016b). Another important feature of the irreversible nucleated growth model is that the end state polymer distribution attains a stationary set of values at the same instant as the polymer mass end state, i.e. only the left-hand side equilibrium in Eq. 14 is operative and therefore no slow rearrangement of the distribution takes place (Bridstrup and Yuan 2016; Hall 2003; Hall and Minton 2004).

We considered two different cases of the irreversible nucleated growth model with regard to the turbidimetric transform. The first case (Fig. 8) explores the effects of different fibre geometry upon the turbidity signal. To examine this, four different examples of fibre radii (4, 6, 8 and 10 nm) are considered, with all cases following identical growth kinetics (Fig. 8a,b). Due to volume conservation requirements, the thinner fibres lengthen faster (Fig. 8c), yet it is the shorter, thicker fibres that show the greatest extent of turbidity (Fig. 8d). With respect to this point, we note that relatively short changes in fibre dimension can effect a large change in the recorded turbidity (Fig. 8d—roughly fivefold for the 4 vs. 10 nm case). The second case considered for the irreversible nucleated growth scheme (Fig. 9) involves examination of the effects of slow to fast nucleus production on the chemical kinetics and accompanying turbidity development of a fibre with fixed geometry (R_A_ = 6 nm). Faster nucleation is known to produce a greater number concentration of smaller (Fig. 9b) and shorter (Fig. 9c) amyloid (Lomakin et al. 1996). Interestingly, as the fibre length falls below a limit of ∼2λ, the corresponding turbidity value, taken as reflecting asymptotic extent, also falls (Fig. 9d) despite there being the same total amount of monomer in amyloid form for all cases of the nucleation rate. Such a decrease in turbidity for very short fibres was first described and theoretically rationalized for microtubule fibre formation (Berne 1974; Gaskin et al. 1974). This phenomenon was later re-examined (Hall and Minton 2005) specifically for the case of microtubules and recently further developed in relation to amyloid and amorphous growth (Hall et al. 2016a).

### Nucleation–growth with fibre breakage

In this mechanism fibres break—both internally, to produce two new fibres, and at their extremities, to release non-amyloid monomers (Hall and Edskes 2004; Masel et al. 1999). The consequences of fibre breakage on the progression of amyloid kinetics have been considered from a number of different perspectives (Hall and Edskes 2004; Masel et al. 1999; Smith et al. 2006; Tanaka et al. 2004), with a detailed model of the potential effects of fibre breakage on amyloidosis-related disease progression being potentially the most important (Hall and Edskes 2009, 2012). With regard to this last point, the importance of both fibre breakage rate and fibre size distributions to aggregate cytotoxicity was demonstrated using a cell culture model (Xue et al. 2009). More recently, Nicoud et al. (2015) have considered further complicating effects upon amyloid growth kinetics associated with potential position dependence of fibre breakage. In the consensus model presented in Table 2, we have reduced Eq. 12a, b to a more tractable form by assuming that all monomer to monomer bonds within the amyloid fibre can break at a rate governed by the first-order rate constant b_A_ (i.e. b_i,j_ = b_A_ for all i, j). As per the irreversible nucleated growth model, all second-order association reactions, in which at least one of the species is assumed to be a monomer, are governed by rate constants f_N_ and f_A_, depending upon the size of the reactants. A summation of the set of rate equations describing the growth and loss of all species greater than the monomer produces the set of rate equations described in Table 2 (Hall and Edskes 2009; Smith et al. 2006). Depending upon the rate of internal fibre breakage, the collapse of the polymer size distribution may be either temporally coupled or decoupled from the kinetics of growth of the polymer mass (Hall and Edskes 2012). An important consequence of this mode of amyloid growth is that the end-point size distribution will always approach—albeit often extremely slowly—the critical nucleus size (Hall and Edskes 2009).

Three different rates of intrinsic fibre breakage (b_A_ = 0 s^−1^, b_A_ = 1 × 10^−9^ s^−1^ and b_A_ = 1 × 10^−8^ s^−1^) were simulated using the consensus reversible fibre growth model (shown in Table 2). Larger values of b_A_ were found to speed up the incorporation of monomer into the amyloid form (Fig. 10a) (Hall and Edskes 2004, 2009). Due to the relatively low values selected for the breakage rate constants, a very slow relaxation of the fibre distribution is seen (Fig. 10b, c). For constant fibre geometry (R_A_ = 6 nm), we note that fibre breakage produces non-ideal turbidimetric kinetic profiles, exhibiting a decrease in the asymptotic extent of turbidity as the fibre length falls below the ∼L > 2λ limit (Fig. 10d).

### Fibre end-to-end association

The joining of shorter fibres to form longer ones has been directly observed in some amyloid systems (Binger et al. 2008). Based on theoretical predictions (relating to differences in number concentration^22^ and intrinsic orientation effects related to the likelihood of two fibre ends meeting (Hill 1983; Pallitto and Murphy 2001), the rate constant governing longitudinal fibre/fibre association (*f*_*i,j*_) is assumed to be much smaller than the fibre/monomer association rate constant (*f*_*i,1*_). As such, the kinetics of fibre annealing is likely to be relatively slow and decoupled from the (relatively) faster kinetics of the monomer/polymer mass reaction. In terms of the equilibrium described by Eq. 14, fibre joining likely exhibits a slow redistribution phase. In our consensus model, the rate of joining between any two fibres is specified by a single rate constant, f_JEE_, such that f_JEE_ << f_A_.

Three cases of fibre joining rate were simulated using the consensus fibre end-to-end joining model shown in Table 2. All simulated cases had the same fibre width, with R_A_ = 6 nm. Following the asymptotic relation predicted for the transmission form factor (Q) for rods (Table 1; Fig. 6), the turbidity is effectively blind to changes in length brought about by joining. In a different kinetic regime (not explored here), fibre joining could significantly influence the turbidimetric profile if it occurred between very small fibres (Hall and Minton 2005) having a length smaller than the L > 2λ limit, (above this limit, specific turbidity becomes effectively independent of fibre length (Berne 1974; Buitenhuis et al. 1994; Hall et al. 2016a).

### Fibre lateral association

Despite widespread descriptions of laterally associated ‘mature fibres’ (Fitzpatrick et al. 2013; Kanno et al. 2005; Ridgley and Barone 2013; Woolfson and Ryadnov 2006; Yamaguchi et al. 2005) and semi-ordered aggregation of amyloid to form spherulites (Krebs et al. 2004a; Ruth et al. 2000), there is a general dearth^23^ of experimental and theoretical studies which have considered the effect of lateral association on amyloid growth kinetics. Three different mechanistic possibilities exist for the production of laterally associated fibres. The first involves heterogeneous nucleation of a new fibre on the surface of a pre-existing amyloid fibre (Jeong et al. 2013; Padrick and Miranker 2002); the second involves lateral association taking place while the fibres are small, with subsequent extension of each growing end of the conjoined fibres (Ghosh et al. 2010; Pallitto and Murphy 2001); the third mechanistic option involves self-association of already formed protofibrils with lateral fibre association governed by rate constants much smaller than the rate constants governing the monomer/polymer mass equilibrium (Eq. 14)(Ghosh et al. 2010; Pallitto and Murphy 2001) i.e. (f_i,j_)_LAT_ << f_i,j_. There is a great deal of complexity in the simulation of any of these three cases. To provide a benchmark kinetic description of the effect of fibre lateral growth, we have opted to simulate a kinetic case that is similar in form to the preceding fibre end-to-end joining model. For simplicity we have limited this to lateral protofibril addition governed by a rate constant f_JLA_, with the level of association fixed to the stage of lateral dimer, denoted as A2 (Table 2). Such a kinetic description dictates a slow association phase in which the polymers become progressively thicker on average (Fig. 12). One potentially misleading assumption in the formulation of the equation set describing lateral association (Table 2) is the subsuming of a multitudinous array of possible lateral associations (involving partial off-centre overlap of fibres) into a single mechanistic path describing centre-to-centre alignment of the fibres. Such off-centre association, or indeed point contact formation, may be responsible for the formation of either extremely long fibres or the birds’ nest-type clusters of fibres often seen in amyloid plaques (Merz et al. 1983; Wisniewski et al. 1989) and in ultra-microscope images (Ban et al. 2003; Mishra et al. 2011; Ogi et al. 2014).

### Amyloid versus amorphous growth

 When there is no orientation or configurational requirement to the association reaction, the internal structure of the aggregate will lack positional order, resulting in the formation of an amorphous product (Bennett 1972; Yoshimura et al. 2012; Zurdo et al. 2001) (Table 2). The production of such amorphous aggregates has been observed in many amyloid-forming systems and often complicates simple interpretation of the reaction. Hall et al. (2015) and Adachi et al. (2015) have treated the case of amyloid growth in competition with amorphous aggregate using a kinetic rate scheme that treated the rate of growth and breakage of all species in an explicit fashion. Here we produce example simulations describing the competition between the amyloid and amorphous aggregate based on a fixed-seeded growth model (Naiki et al. 1997). In this mechanistic format, growth proceeds reversibly, for both amorphous and amyloid aggregate types, from a fixed (i.e. non-dissociable) seed species of degree <i>_t=0_. Here we consider growth and shrinkage as occurring via monomer addition and monomer loss only, with no fragmentation or spontaneous nucleation allowed. For amyloid growth this implies the following boundary conditions:f_i,1_ = f_A_ for <i_A_> ≥ <i_A_>_t=0_, else all f_i,1_ = 0 and b_i-1,1_ = b_A_ for <i_A_> > <i_A_>_t=0_, else b_i-1,1_ = 0; for amorphous aggregate growth, the following boundary conditions are implied: f_i,1_ = f_G_ for <i_G_> ≥ <i_G_>_t=0_, else all f_i,1_ = 0 and b_i-1,1_ = b_G_ for <i_G_> > <i_G_>_t=0_, else b_i-1,1_ = 0. Hall and coworkers explored the case where amyloid is more thermodynamically stable than the amorphous aggregate, but slower to initially form (Hall et al. 2015). This study, along with work by Adachi et al. (2015), highlighted a potential dependence of the time-scale of amyloid formation on the dissociation rate of the amorphous species.

To explore the effects of competing amorphous growth in extension to that performed previously (Adachi et al. 2015; Hall et al. 2015), we simulated three cases of competition between amorphous aggregate and amyloid using the consensus model presented in Table 2 (Fig. 13). The rate constants were selected to ensure that the amyloid was ultimately more stable, in the thermodynamic limit, than the amorphous aggregate in all cases (Fig. 13a–c). Despite this preponderance for amyloid growth, even relatively small amounts of amorphous aggregate can significantly distort the resultant turbidimetric kinetic profile (Fig. 13d, black lines).

### Amyloid versus amyloid growth—two strains in competition

 Kinetic competition between two types of amyloid has not yet, to the best of our knowledge, been quantitatively investigated, but it is known to be an important feature of real-case amyloid growth in which different polymorphic strains are observed (Hall and Edskes 2004; Paravastu et al. 2008; Petkova et al. 2005; Tanaka et al. 2004). To explore this behaviour in isolation, we modelled two types of amyloid fibre, both competing for the same monomer pool, using a fixed seeded reversible growth scheme virtually identical to that adopted for the amorphous versus amyloid case (reported in the preceding subsection) with the same requirement that fibres grow or shrink via monomer addition or loss only^24^ (Table 2). The two fibre types are characterized by different radii (R_A#1_ and R_A#2_). Growth for the type #1 fibre is defined by the following set of boundary conditions: f_i,1_ = f_A#1_ for <i_A#1_> ≥ <i_A#1_>_t=0_, else all f_i,1_ = 0 and b_i-1,1_ = b_A#1_ for <i_A#1_> > <i_A#1_>_t=0_, else b_i-1,1_ = 0; growth for type #2 fibres is defined by f_i,1_ = f_A#2_ for <i_A#2_> ≥ <i_A#2_>_t=0_, else all f_i,1_ = 0 and b_i-1,1_ = b_A#2_ for <i_A#2_> > <i_A#2_>_t=0_, else b_i-1,1_ = 0.

Two different cases of competitive fibre growth were explored (Fig. 14). The first involved the situation where a relatively thin type #1 fibre (R_A#1_ = 5 nm) outcompeted a slightly thicker type #2 fibre (R_A#2_ = 6 nm) for monomer resources (Fig. 14a–c). The second case involved the reverse situation, in which the slightly wider type #2 fibres eventually outcompeted the thinner type #1 fibres for monomer (Fig. 14a–c). As can be noted (Fig. 14d), even relatively subtle differences in dimensions between the dominant and non-dominant fibre types will impart significant non-ideality to the resultant kinetic profile recorded via turbidity (dotted black or cyan lines in Fig. 14d).

## Towards the future

Concern over the interpretation of potentially non-linear signal response is a repeating and important theme in science (Araujo 2009). Pursuing this line of investigation, we have focussed on literature capable of informing the reader about the cause and effect relationship between protein aggregates and the turbidity generated by them in solution. Using a slightly non-conventional review format we have combined published transforms with consensus kinetic models to produce ‘review-data’, cutting out the requirement for worded descriptions otherwise necessary for synthesizing arguments from multiple information streams.

As can be noted from Fig. 6d, straight rod-like fibres possessing a common width should produce a signal that is linearly proportional to the mass of monomer in aggregate form when the fibres are long in relation to the wavelength (Hall et al. 2016a) (or in the words and symbols of this review, τ ∝ C_M→A_ when L_A_ > 2λ). Outside of this limit a linear relationship will not necessarily hold and should be either (1) investigated experimentally (e.g. Borgia et al. 2013; O’Nuallain et al. 2006), (2) compared against results gained from an orthogonal technique (Li et al. 2009; Nilsson 2004) or (3) examined using some of the theoretical and simulation-based tools highlighted in this review. A fourth option, previously explored by a number of researchers, involves (4) experimental interrogation of the wavelength dependence of the turbidity (Camerini-Otero and Day 1978; Wallach et al. 1961) to gain clues about dominant aggregate sub-types (Andreu and Timasheff 1986; Garcia-Lopez and Garcia-Rubio 2008; Garcia-Lopez et al. 2006; Hall and Minton 2005; Korolevskaya and Khlebtsov 2010; Mahler et al. 2005; Moody et al. 1996; Silver and Birk, 1983)). Advances in computer power make re-visitation of this multi-wavelength approach an attractive area of current and future research (Mroczka and Szczuczynski 2010; Penzkofer et al. 2007; Shmakov 2014).

By far, the major focus of amyloid research remains its association with diseases, collectively termed as amyloidosis, which are all characterized by the deposition of large amounts of amyloid into various organs and tissues of the human body (Symmers 1956; Pepys, 2001; Walker and Jucker 2015). Potential non-linear effects, which complicate the interpretation of the turbidity signal, become very important when turbidity is used as an assay for anti-amyloid drug screening (Anzai et al. 2016; Doig et al. 2004; Dolado et al. 2005; Necula et al. 2007; Sant’Anna et al. 2016). In such cases, the use of an orthogonal technique, such as Congo Red or Thioflavin T dye binding, as a control experimen, should go a long way towards preventing spurious conclusions from being drawn.

From its original negative association with disease, amyloid has since been found to play potentially beneficial roles in non-disease-related areas. Two such positive manifestations of amyloid include (1) the discovery of its role in maintaining the normal biological state as 'functional amyloid' (Greenwald and Riek 2010) and (2) amyloid’s potential in biosynthetic applications (Mitraki 2010; Raynes and Gerrard 2013). In this latter role, amyloid’s nanometer-scale dimensions (Xu et al. 2016), its inherent capacity for autonomous self-assembly (Lee et al. 2015; Sasahara et al. 2010) and the desirable material properties of the nanofiber product (Paul et al. 2016) all highlight the potential usefulness of amyloid as a ‘building block’ in nanotechnology applications (Rodina 2012). Due to their simplicity, turbidity assays will continue to be the ‘go to’ technique for monitoring amyloid formation across these disparate research areas. A greater familiarity with the principles of the turbidimetric technique will undoubtedly facilitate research progress throughout the wider community. Hopefully this review has helped to decrease the foggy nature of turbidity, allowing some metaphorical blue skies to be seen through the cloud.

## Footnotes

1 Latin: amylum; Greek: amylon *meaning* starch or ground meal

2 Differential diffraction is dependent upon the relative rotation of the fibre along the cylindrical coordinate.

3 For a reversible process considered in the thermodynamic limit, this can be a consequence of the statistical/entropic factors associated with a multi-step reaction pathway (Hall and Minton 2002, 2004). For both reversible and irreversible processes considered in the kinetic limit, heterogeneity in reaction products may also be caused by spatial fluctuations in concentration, molecular fluctuations in configuration and/or energetic fluctuations brought about through collisions with the solvent.

4 Lateral association of fibres is an example of a hierarchical mode of aggregation, in which multiple thinner fibres (sometimes termed protofibrils) laterally self-associate to form thicker fibres designated as mature fibres (Makin and Serpell 2002). Other types of hierarchical aggregation products are also observed (Krebs et al. 2004; Ridgley and Barone 2013).

5 Mesoscopic physics is concerned with materials of an intermediate length, i.e. from the nanometer to the micrometer range, which roughly spans the experimental space between the atomic and the macroscopic. Mesoscopic models do not display atomic/molecular features but tend to be based on approximate regular structures, such as spheres and hard convex/cylindrical bodies.

6 Indirect estimates of aggregate shape can also be made from model-based deconvolution of hydrodynamic (Lomakin et al. 1996; (Rogers et al. 2005) or molecular weight measurements (Pallitto and Murphy 2001; Nichols et al. 2002).

7 In the preparation of our review we noticed that the corresponding equation of Hall (2012) has a typographical error with regard to bracket placement.

8 To a first approximation υ can be taken as having a constant value of ∼0.73 ml/g although Lee and co-workers (Lee et al. 2009) have shown that this value may decrease in the amyloid state, i.e. amyloid may be more tightly packed than normal globular proteins.

9 Amorphous means an aggregate in which the monomeric units constituting the aggregates possess no regular internal structure (Bennett 1972).

10 In a similar fashion, most solid state NMR structures require determination of an average density per unit length by scanning TEM experiment (e.g. Petkova et al. 2005).

11 As a simple example, we might consider the histogram limits as referring to aggregate degree i, such that we consider the bin elements defined from i → i + Δi.

12 Inelastic light scattering refers to the case where some energy is absorbed, donated or dissipated by the molecule with subsequent change in wavelength between incident and scattered radiation.

13 As issues related to high concentration can be effectively solved experimentally by either using a smaller path length or by serial dilution of the solution prior to measurement, we focus in this review on the effects related to particle size and shape complexity. With this point made, we note that the dilute limit refers to the concentration range at which one may neglect significant re-scattering of the scattered light by other aggregate particles in solution back into the collimated detection pathway. This re-scattering effect by other molecules in solution is sometimes called external interference. We refer the reader to work describing the effect of external interference on scattering encountered in the high concentration limit based on virial expansions of the turbidity in terms of solute concentration (Dhont 1983; Minton 2007). Such factors will be important to consider if the turbidimetric technique is be used to study aggregation in near cell-like conditions such as those employed in macromolecular crowding studies (Hall and Minton, 2003; Hall, 2002; Hall and Dobson, 2006; Hall 2006).

14 Two important points to note here are (1) Q_SPHERE_ in the Mie region was calculated using the AD approximation and (2) Q_ROD_ in the Mie region was calculated by extending the asymptotic form calculated by the RGD approach. Although not exact, this assumption has been calculated to induce an error of <15 % in the estimation of scattering of non-polarized light from randomly oriented fibres extending in length beyond the RGD limit up to an (effectively) infinite length (Bishop 1989; (Buitenhuis et al. 1994).

15 If aggregates are composed of multiple isomeric forms the rate constants become dependent upon the isomeric state and a new dimension must be introduced into the specification of the aggregate and rate constants.

16 Corrected for statistical and stoichiometric factors.

17 Also known as templated-growth, nucleated-crystal growth or helical polymerization (Oosawa and Asakura 1975).

18 As linear polymers of amino acids, proteins potentially have available to them a very great number of possible internal configurations of the polymer chain. Internal bonding preferences for solvent and self often limit this range of possibilities, leading to a single structural state known as the folded state. Under conditions of elevated temperature, the presence of a chemical denaturant or other structure-deforming species, the protein can be induced to unfold.

19 Approximation of the distribution by the average prior to estimation of the turbidity will introduce another type of error into the process.

20 In the modern day computer-based numerical integration of ODE sets lessens the requirement for the determination of analytical solutions that may be based on further mathematical approximations (in addition to the already existing chemical approximations).

21 Interestingly, with regard to this point, it was shown that a nucleation event could be generated for a linear polymerization process based solely on consideration of the conformational entropy of the unfolded protein—that is an entropic nucleus (Hall et al. 2005); Hall and Hirota 2009).

22 i.e. the monomer number concentration is higher than the fibre number concentration nearly throughout the monomer to polymer mass equilibrium.

23 With some exceptions (Ghosh et al. 2010; Pallitto and Murphy 2001).

24 We note that the general behaviour outlined for the two competitive cases (‘Amyloid vs. amorphous growth’ and ‘Amyloid versus amyloid growth—two strains in competition’) are also applicable to the description of the growth and dissolution of different crystal forms (Levi and Kotrla 1997).
